# Intragenic suppressor mutations of the *COQ8* protein kinase homolog restore coenzyme Q biosynthesis and function in *Saccharomyces cerevisiae*

**DOI:** 10.1371/journal.pone.0234192

**Published:** 2020-06-01

**Authors:** Agape M. Awad, Anish Nag, Nguyen V. B. Pham, Michelle C. Bradley, Nour Jabassini, Juan Nathaniel, Catherine F. Clarke

**Affiliations:** Department of Chemistry and Biochemistry, and the Molecular Biology Institute, University of California, Los Angeles, California, United States of America; Tulane University Health Sciences Center, UNITED STATES

## Abstract

*Saccharomyces cerevisiae* Coq8 is a member of the ancient UbiB atypical protein kinase family. Coq8, and its orthologs UbiB, ABC1, ADCK3, and ADCK4, are required for the biosynthesis of coenzyme Q in yeast, *E*. *coli*, *A*. *thaliana*, and humans. Each Coq8 ortholog retains nine highly conserved protein kinase-like motifs, yet its functional role in coenzyme Q biosynthesis remains mysterious. Coq8 may function as an ATPase whose activity is stimulated by coenzyme Q intermediates and phospholipids. A key yeast point mutant expressing Coq8-A197V was previously shown to result in a coenzyme Q-less, respiratory deficient phenotype. The A197V substitution occurs in the crucial Ala-rich protein kinase-like motif I of yeast Coq8. Here we show that long-term cultures of mutants expressing Coq8-A197V produce spontaneous revertants with the ability to grow on medium containing a non-fermentable carbon source. Each revertant is shown to harbor a secondary intragenic suppressor mutation within the *COQ8* gene. The intragenic suppressors restore the synthesis of coenzyme Q. One class of the suppressors fully restores the levels of coenzyme Q and key Coq polypeptides necessary for the maintenance and integrity of the high-molecular mass CoQ synthome (also termed complex Q), while the other class provides only a partial rescue. Mutants harboring the first class of suppressors grow robustly under respiratory conditions, while mutants containing the second class grow more slowly under these conditions. Our work provides insight into the function of this important yet still enigmatic Coq8 family.

## Introduction

Coenzyme Q (also termed ubiquinone, CoQ or Q) serves as a vital redox active lipid and antioxidant molecule necessary for energy production [1,2]. Q is composed of a fully substituted benzoquinone ring and a polyisoprenoid tail whose length is species dependent [3]. *Homo sapiens* produce Q_10_ with a decaprenyl tail, *Escherichia coli* produce Q_8_, and *Saccharomyces cerevisiae* produce Q_6_. A primary role of Q in the inner mitochondrial membrane is to serve as a reversible electron and proton carrier [4]. Q accepts electrons and protons from Complexes I and II in the respiratory electron transport chain, and QH_2_ (ubiquinol or reduced CoQH_2_) donates electrons and protons to Complex III [5]. Q also functions as an electron acceptor in other biochemical pathways, such as pyrimidine synthesis, sulfide oxidation, and fatty acid β-oxidation [4,6,7]. Human Q_10_ deficiencies often affect multiple organ systems, including the central and peripheral nervous systems, kidney, skeletal muscle, heart, and sensory systems [6]. Mouse knockout studies show that a complete lack of Q is embryonic lethal [8]. In contrast, yeast mutants lacking Q_6_ are unable to grow on nonfermentable carbon sources, but are able to grow on fermentable carbon sources, because ATP is generated via substrate-level phosphorylation.

**Fig 1:**
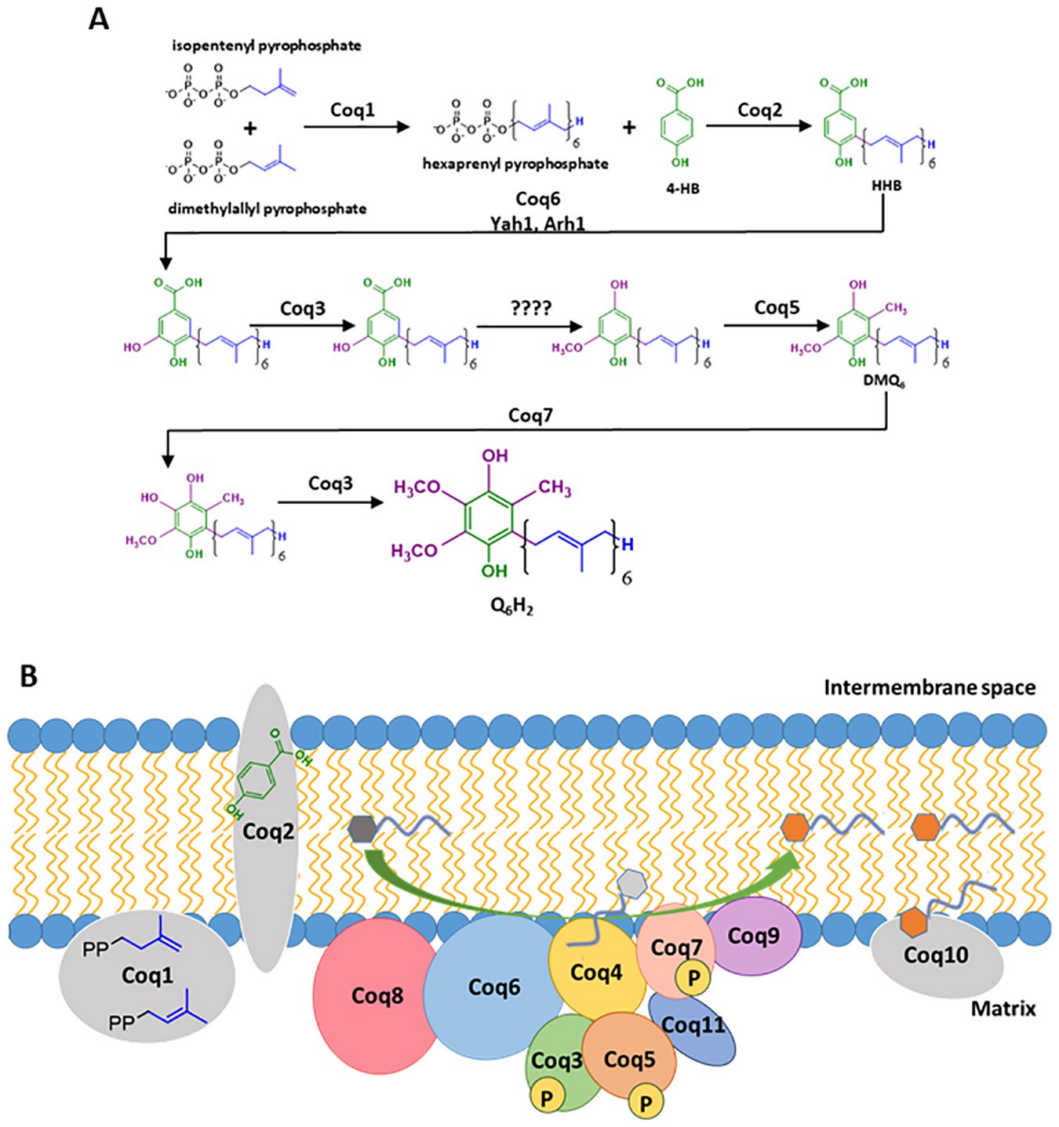
Coenzyme Q biosynthetic pathway in *S*. *cerevisiae* and the formation of the high-molecular mass CoQ synthome. *A*, The pathway for the enzymatic formation of Q_6_ in yeast, starting with 4-hydroxybenzoic acid as the ring precursor. Questions marks indicate unknown steps involved in the decarboxylation and hydroxylation of the intermediate(s) leading to reduced coenzyme Q_6_H_2_. *B*, The schematic of a high-molecular mass complex in yeast, termed the CoQ synthome. Coq1, Coq2, and Coq10 polypeptides are not observed as members of the high-molecular mass complex. Coq1 and Coq2 produce HHB (designated by the dark gray hexagon). This early CoQ-intermediate is converted by subsequent action of Coq6 and other Coq polypeptides to an essential lipid component of the CoQ synthome (shown as a light gray hexagon in association with Coq4). Coq8 physically associates with Coq6 and is an ancient atypical kinase, thought to be responsible for the regulatory phosphorylation of Coq3, Coq5 and Coq7 and/or ATPase activity. Q_6_, the product of the CoQ synthome, is designated by the orange hexagon.

Fourteen nuclear encoded mitochondrial proteins are necessary for the efficient production of Q_6_ in *S*. *cerevisiae* (Fig 1A) [9,10]. Coq1 is responsible for the synthesis of hexaprenyl diphosphate, and Coq2 attaches the hexaprenyl group to 4-hydroxybenzoic acid (4-HB). Other Coq polypeptides work to catalytically modify the head group via decarboxylation, hydroxylation, and methylation steps, in order to produce QH_2_. Several of the Coq proteins involved in the synthesis of Q_6_ in *S*. *cerevisiae* associate in a high molecular mass complex termed the CoQ synthome, and localize to the inner mitochondrial membrane on the matrix side (Fig 1B) [9,11]. Coq4 serves as a scaffolding protein and is the central organizer of the CoQ synthome [12]. Many of the other Coq polypeptides, including Coq3, Coq5, Coq6, Coq7, Coq8, Coq9, and Coq11 are partner proteins required for CoQ synthome stability, assembly, or enzyme activity [9,11].

Coq8 is a member of the ancient protein kinase-like (PKL) UbiB family of proteins, as well as a member of the superfamily of putative atypical protein kinases termed “ADCKs” (aarF-domain containing kinases) [13]. The UbiB PKL family comprises nearly a quarter of all known microbial PKL proteins, is involved in isoprenoid lipid synthesis, and is necessary for the aerobic production of Q [14]. In humans, there are five UbiB-like homologs, termed ADCK1—ADCK5; deficiencies in ADCK1, ADCK2, ADCK3 or ADCK4 have been implicated in various diseases. ADCK3 (COQ8A) and ADCK4 (COQ8B) are co-orthologs of yeast Coq8, since each can rescue the yeast *coq8* null mutant [15,16]. Patients harboring mutations in *ADCK3* develop cerebellar ataxia [17,18], while patients with mutations in *ADCK4* develop steroid resistant nephrotic syndrome [16,19]. Both types of patients have significantly decreased levels of Q_10_. Recently, *ADCK2* haploinsufficiency was observed to cause liver dysfunction, impaired fatty acid oxidation, and mitochondrial myopathy in skeletal muscle in one patient and in a mouse model [20].

Structural and biochemical studies of human COQ8A revealed an atypical kinase-like fold, and ATP and ADP were bound in a divalent cation-dependent manner [13]. Sequence alignments identified nine conserved PKL subdomains [13]. Analyses of yeast *coq8* mutants with mutations present within the PKL motifs suggest that these conserved motifs are essential for Q biosynthesis [13,15]. Creation of an ATP analog-sensitive version of yeast Coq8 showed that chemical inhibition with targeted ATP-based inhibitors could be used to rapidly induce a respiratory deficient phenotype and depletion of Q_6_ [21]. These studies indicate that ATP binding and hydrolysis is essential to the function of Coq8 and COQ8A in the biosynthesis of Q. An atypical Ala-rich loop defines the PKL-motif 1 of COQ8A and replaces the Gly-rich loop present in the active site of canonical protein kinases [13]. The mutation of a single alanine residue (A339) to a glycine within the Ala-rich loop of kinase-like motif 1 elicited enhanced autophosphorylation of COQ8A, but decreased Q production. The A339 of human COQ8A corresponds to A197 of yeast Coq8; the Coq8-A197V mutation was shown to abolish Q_6_ production in yeast [15].

Several Coq polypeptides are shown to be phosphorylated in a Coq8-dependent manner—particularly Coq3, Coq5, and Coq7—although there is no direct evidence that Coq8 is the kinase that is responsible for this phosphorylation [15]. Instead, mammalian COQ8A and COQ8B, and yeast Coq8, have been proposed to function as ATPases or small molecule kinases, rather than as canonical protein kinases [22]. In this capacity Coq8 may somehow act to assist the formation or stability of the CoQ synthome. Coq8 and its ATPase activity are also required for the organization of the CoQ synthome into puncta or discrete domains that occur at contact sites between the mitochondria and ER [23,24]. Coq4, Coq7, and Coq9 polypeptides are often used as sensitive indicator polypeptides of the CoQ synthome [12,25]. Overexpression of Coq8 augments the steady state levels of these indicator Coq polypeptides and stabilizes the CoQ synthome [12,25,26].

In this study, we employed a yeast *coq8*-3 mutant harboring an A197V mutation that was previously characterized as “Q-less” and unable to grow on rich medium containing glycerol (YPG) [15]. We recovered spontaneous revertants that acquired the ability to grow on YPG. Surprisingly, characterization of the revertants reveals that each contains a secondary mutation within the *COQ8* gene, and thus are intragenic suppressors. Overall, we identify and characterize three novel point mutations that appear to be highly influential in the mode of action of Coq8 and its contribution to Q production and CoQ synthome stability.

## Materials and methods

All reagents were obtained commercially from Fisher Scientific unless otherwise specified.

### Yeast strains and culture conditions

The *S*. *cerevisiae* strains used in this study are listed in Table 1.

**Table 1:** Genotypes and sources of yeast strains.

Strain	Genotype or description	Source
W303-1A	MAT **a** *leu2-3*,*112 trp1-1 can1-100 ura3-1 ade2-1 his3-11*,*15*	R. Rothstein^a^
C183	MAT α *met6 coq8-3*	[27]
W183-2A	MAT **a** *his3-1*,*15 trp1-1 ura3-1 coq8-3*	[15]
JM6	MAT **a** *his4 rho*^*0*^	J.E. McEwen^b^
JM8	MAT α *ade1 rho*^*0*^	J.E. McEwen^b^
W303*Δcoq8*	MAT **a** *ade2-1 his3-1*,*15 leu2-3*,*112 trp1-1*, *ura3-1 coq8::HIS3*	[28]
NP-183A	MAT **a** *leu2-3*,*112 trp1-1 can1-100 ura3-1 ade2-1 his3-11*,*15 coq8-3*	This work
NP-183AL	MAT **a** *trp1-1 can1-100 ura3-1 ade2-1 his3-11*,*15 coq8-3*	This work
NP-183B	MAT α *leu2-3*,*112 trp1-1 can1-100 ura3-1 ade2-1 his3-11*,*15 coq8-3*	This work
NP-183BH	MAT α *leu2-3*,*112 trp1-1 can1-100 ura3-1 ade2-1 coq8-3*	This work
Rev-AL	MAT **a** *trp1-1 can1-100 ura3-1 ade2-1 his3-11*,*15 coq8-3 SupRA*	This work
Rev-BL	MAT **a** *trp1-1 can1-100 ura3-1 ade2-1 his3-11*,*15 coq8-3 SupRB*	This work
Rev-CL	MAT **a** *trp1-1 can1-100 ura3-1 ade2-1 his3-11*,*15 coq8-3 SupRC*	This work
Rev-DL	MAT **a** *trp1-1 can1-100 ura3-1 ade2-1 his3-11*,*15 coq8-3 SupRD*	This work
Rev-EL	MAT **a** *trp1-1 can1-100 ura3-1 ade2-1 his3-11*,*15 coq8-3 SupRE*	This work
NPD-NP	diploid produced from NP-183BH × NP-183AL	This work
NPD-A	diploid produced from NP-183BH × Rev-AL	This work
NPD-B	diploid produced from NP-183BH × Rev-BL	This work
NPD-C	diploid produced from NP-183BH × Rev-CL	This work
NPD-D	diploid produced from NP-183BH × Rev-DL	This work
NPD-E	diploid produced from NP-183BH × Rev EL	This work

^a^ Dr. Rodney Rothstein, Department of Human Genetics Columbia University

^b^ Dr. Joan E. McEwen

All strains were derived from the W303 genetic background. Liquid yeast culture medium was prepared as described [11] and included YPD (2% glucose, 1% yeast extract, 2% peptone), YPGal (2% galactose, 0.1% glucose, 1% yeast extract, 2% peptone), and YPG (3% glycerol, 1% yeast extract, 2% peptone). Yeast plate medium was prepared by adding 2% Agar (Bacto) to the above mentioned liquid media. Yeast strains were stored in 30% (v/v) sterile glycerol at −80 °C. Prior to each experiment, the corresponding strains were plated on to YPD plates, incubated at 30 °C for two to three days, and the plates with yeast colonies were stored at 4 °C for up to two weeks.

### Use of CRISPR/Cas9 to generate NP-183A and NP-183AL

pCAS plasmid was obtained from Addgene (plasmid #60847) [29] (Table 2). NP-183A and NP-183AL strains harboring the *coq8-3* point mutation were prepared via standard yeast transformation protocol [30] with inclusion of 1 μg of the pCas9 plasmid along with the standard repair DNA for homologous recombination. The *COQ8* sequence in proximity of the *coq8-3* point mutation (C590T encoding A197V) was designed following a Protospace Adjacent Motif (PAM) sequence. A 60-basepair repair double stranded DNA that emulated the *coq8-3* point mutation was prepared through restriction free cloning with the QuikChange II Site-directed mutagenesis kit (Agilent, Santa Clara, CA). The newly desired plasmid and the repair DNA were used to transform W303-1A. Transformants were plated on YPD with G418 and incubated at 37 °C for one to two days to activate the Cas9 enzyme. Colonies observed on YPD with G418 were isolated and grown on YPD at 30 °C. The *COQ8* gene sequence was verified through sequencing and over the region of the *COQ8* gene, NP-183A and NP-183AL (Table 1) each contained just the *coq8-3* mutation.

**Table 2:** Plasmid constructs used in this study.

Plasmid	Construct Description	Copy Number	Source
pCAS	Expresses *S*. *pyogenes* Cas9 plus a HDV ribozyme-sgRNA for genome editing in yeast	Multi copy	[29]
p3HN4	Yeast ABC1/COQ8	Low copy	[31]
p4HN4	Yeast ABC1/COQ8	Multi copy	[32]
plc-Coq8-A197V	Yeast ABC1/COQ8 with Coq8-A197V	Low copy	This work
plc-Coq8-S232N	Yeast ABC1/COQ8 with Coq8-S232N	Low copy	This work
plc-Coq8-A197V/S232N	Yeast ABC1/COQ8 with Coq8-A197V and S232N	Low copy	This work

### Yeast mating type switch

Mating type switch for NP-183A was performed with the pGal-HO plasmid as described [33]. Single colonies were selected and tested for intact mitochondrial genome by mating with JM6 and JM8 *rho*^*0*^ tester strains (Table 1). The rho test also provided verification of the mating type switch.

### Yeast sporulation and tetrad dissection

Diploid cells were induced to sporulate and tetrads dissected as described [34]. Respiratory defective yeast diploid strains were transformed with p3HN4 expressing the wild-type *COQ8* gene prior to sporulation. Treatment with 5-fluoro-orotic acid (5-FOA) was used to remove p3HN4 from resulting haploid progeny [34].

### Site-directed mutagenesis

Site-directed mutagenesis was performed with the QuikChange Lightning mutagenesis kit and the XL10 transformation according to the manufacturer’s directions (Agilent, Santa Clara, CA).

### Purification of mitochondria

Yeast cells were grown in 5 ml of YPGal plus 0.1% dextrose precultures and inoculated into 600-ml YPGal plus 0.1% dextrose cultures for overnight growth in a shaking incubator (30 °C, 250 rpm). Cells were harvested at an A_600nm_ of 3.5–4.0, and mitochondria were purified as described [35] and [11]. Protein concentration was measured with a BCA assay using bovine serum albumin as the standard.

### SDS-PAGE and Immunoblot analyses

Protein samples incubated with SDS sample buffer (50 mM Tris-HCl, pH 6.8, 10% glycerol, 2% SDS, 0.1% bromphenol blue, 1.33% β-mercaptoethanol) were separated on 12% Tris-glycine SDS-polyacrylamide gels by electrophoresis [36] for 2 hrs at 135 V followed by transfer to Immobilon-P PVDF membranes (Millipore) at 150 V for 1 hr. Membranes were then blocked overnight in 3% nonfat milk, phosphate-buffered saline (140.7 mM NaCl, 9.3 mM Na_2_HPO_4_, pH 7.4), 0.1% Tween 20 or BSA based blocking buffer for compatibility with LiCor imaging. Membranes were then probed with primary antibodies (Table 3) in 2% nonfat milk, phosphate buffered saline, 0.1% Tween 20 or with antibodies diluted in BSA-based buffer compatible with LiCor imaging. Goat anti-rabbit secondary antibody conjugated to horseradish peroxidase (Calbiochem) was used at 1:10,000 dilutions. Blots were visualized using Supersignal West Pico chemiluminescent substrate or directly via the LiCor system when secondary antibody used was conjugated to a fluorescent signal probe.

**Table 3:** Antibodies used in this study.

Antibody	Working solution	Source
Coq4	1:1000	[37]
Coq7	1:500	[38]
Coq8	1:30 (affinity purified)	[39]
Coq9	1:1000	[39]
Mdh1	1:10,000	Lee McAlister-Henn^a^

^a^Dr. Lee McAlister-Henn, Department of Molecular Biophysics and Biochemistry, University of Texas Health Sciences Center, San Antonio.

### Metabolic labeling of Q_6_ with ^13^C_6_-labeled precursors

In order to assess the content of de novo synthesized Q_6_, yeast strains were grown as described [40]. Briefly yeast strains were incubated overnight in 5 ml of YPD in a shaking incubator (30 °C, 250 rpm) and diluted to an A_600nm_ of 0.1 in 6 ml of fresh YPD the next morning. The cultures were incubated as before to an A_600nm_ of 0.5 (mid-log phase) and subsequently treated with ^13^C_6_-4HB at 10 μg/ml final concentration (ethanol 0.015%, v/v). At designated time periods, cells were harvested by centrifugation at 3000 × *g* for 5 min, from 5 ml aliquots. Cell pellets were stored at −20 °C.

### Analysis of Q_6_ and Q_6_ intermediates

Q_6_ was obtained from Avanti Polar Lipids, Inc. Lipid extraction of cell pellets was conducted as described [40] with methanol and petroleum ether. Prior to extraction Q_4_ was added as the internal standard. To determine the Q_6_ content in yeast strains cultured on YPG plates, the strains were applied to YPG plates and incubated at 30 °C four to five days before harvesting. Cells recovered from the solid plate medium were suspended in YPG liquid medium and the A_600_ determined. Cells were then collected by centrifugation and subjected to lipid extraction. Yeast strains that failed to grow on YPG were subjected to the same analysis, except that a section of the solid plate medium harboring the inoculated yeast cells was excised and subjected to the lipid extraction protocol. This was performed in order to recover cells that were applied to the plate medium but failed to grow. Lipid measurements were performed by HPLC-MS/MS and normalized to total A_600nm_. Prior to mass spectrometry analyses, all samples were treated with 1.0 mg/ml benzoquinone to oxidize hydroquinones to quinones. Mass spectrometry analyses utilized a 4000 QTRAP linear MS/MS spectrometer (Applied Biosystems), and data were acquired and analyzed using Analyst version 1.4.2 and 1.5.2 software (Applied Biosystems). Separation of lipid quinones was performed with a binary HPLC delivery system and a Luna 5μ phenyl-hexyl column (100 × 4.6 mm, 5 μm; Phenomenex). The mobile phase consisted of a 95:5 methanol/isopropyl alcohol solution with 2.5 mM ammonium formate as solution A and a 100% isopropyl alcohol solution with 2.5 mM ammonium formate as solution B. The percentage of solution B was increased linearly from 0 to 5% over 6 min, whereby the flow rate was increased from 600 to 800 μl. Initial flow rate and mobile phase conditions were changed back to initial phase conditions linearly over 3.5 min. Each sample was analyzed using multiple reaction monitoring mode. The following precursor-to-product ion transitions were detected as well as the +17 m/z ammoniated adducts for each of the metabolic products: ^13^C_6_-HHB m/z 553.4/157.0 (ammoniated: 570.4/157.0), ^12^C-HHB m/z 547.4/151.0 (ammoniated: 564.4/151.0), ^13^C_6_-DMQ_6_ m/z 567.6/173.0 (ammoniated: 584.6/173.0), ^12^C-DMQ_6_ m/z 561.6/167.0 (ammoniated: 578.6/167.0), ^13^C_6_-Q_6_ m/z 597.4/203.1 (ammoniated: 614.4/203.1), ^12^C-Q_6_ m/z 591.4/197.1 (ammoniated: 608.4/197.1), and ^12^C-Q_4_ m/z 455.4/197.0 (ammoniated: 472.4/197.0).

### Plate dilution assays

Strains were grown overnight in 5 ml of YPD as described [40] and diluted to an A_600_ of 0.2 in sterile PBS. A 5-fold serial dilution in PBS was performed, after which 2 μl of each dilution (1 X, 5 X, 25 X, 125 X, and 625 X) were spotted onto the designated plate growth medium. The final A_600_ of the aforementioned dilution series are 0.2, 0.04, 0.008, 0.0016, and 0.00032, respectively. The plates were incubated at 30 °C for the designated time periods in days and subsequently imaged.

### PHYRE homology modeling

*S*. *cerevisiae* Coq8 was modeled with the PHYRE2 intensive modeling mode [41]. A 44% identity was obtained compared to PDB ID 4PED, a crystal structure determined for an amino-terminal truncated human COQ8A. The modeled structure lacks the first 32 amino acids of yeast Coq8, predicted to function as the mitochondrial targeting sequence. This sequence is excised upon transport of Coq8 to the mitochondria, resulting in the mature Coq8 polypeptide.

## Results

### Single nucleotide mutations within *COQ8* restore respiratory growth of the Coq8-A197V mutant

A yeast mutant harboring the *coq8-3* allele (see Table 1 for the complete genotype), is respiratory defective, fails to grow on medium with glycerol as the sole carbon source, and lacks Q_6_ [15,27]. The *coq8-3* mutation was identified as A197V, resulting from C590T in the DNA sequence of the *COQ8* gene (Table 4) [15].

**Table 4:** Amino acid and nucleotide substitution of *coq8* alleles.

Strain	Amino acid substitution (nucleic acid mutation)^a^
NP-183AL	A197V (C590T)
Rev-AL	A197V (C590T) and L237P (T710C)
Rev-BL	A197V (C590T) and P220S (C658T)
Rev-CL	A197V (C590T) and S232N (G695A)
Rev-DL	A197V (C590T) and S232N (G695A)
Rev-EL	A197V (C590T) and S232N (G695A)

^a^Position of mutations are relative to the ATG start codon of *COQ8*.

In order to isolate and characterize spontaneous *coq8-3* revertants, we recreated the Coq8-A197V mutation in the W303-1A wild-type genetic background. To accomplish this, the C590T mutation was introduced into the W303-1A genome via CRISPR/Cas9 as described in *Materials and Methods*, and gave rise to yeast strains NP-183A and NP-183AL, that each expressed Coq8-A197V (Table 1). Three independent screens were performed to search for spontaneous revertants, with the acquired ability to grow on YPG plate medium after incubation at 30 °C for three to four weeks. Colonies observed on the YPG screening plates were not always capable of sustained growth when reapplied to fresh YPG plate medium. Thus, colonies were assessed by plate dilution assays prior to being identified as potential revertants.

**Fig 2:**
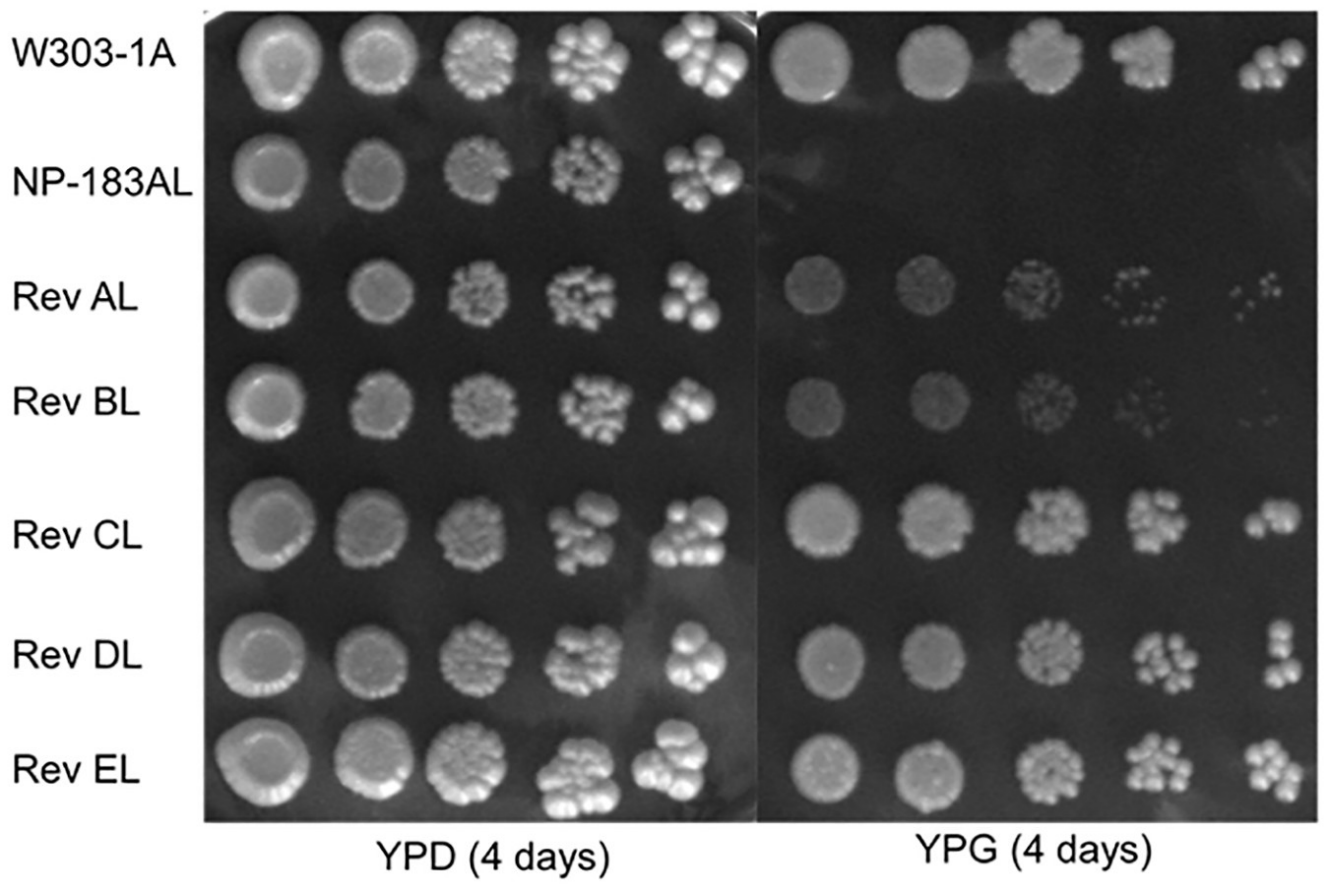
Spontaneous revertants of NP-183AL expressing Coq8-A197V show respiratory growth on YPG plate medium. The designated yeast strains (Table 1) were each grown overnight in 5 mL of YPD, diluted to an A_600nm_ of 0.2 with sterile PBS, and 2 μL of 5-fold serial dilutions were spotted onto the designated plate medium, corresponding to a final A_600nm_ of 0.2, 0.04, 0.008, 0.0016, and 0.00032. Plates were incubated at 30 °C, and growth is depicted after four days. Results shown are representative of two independent biological replicate experiments (see S8 Fig).

This procedure produced five independent revertant yeast strains termed Rev-AL, Rev-BL, Rev-CL, Rev-DL, and Rev-EL that carry suppressor mutations (Table 1). Qualitatively, the robust growth of Rev-CL, Rev-DL and Rev-EL was comparable to WT, while less robust growth was observed for Rev-AL and Rev-BL on YPG (Fig 2). Sequence analyses of the *coq8* locus in each of the five revertant yeast strains showed retention of the parental Coq8-A197V mutation (Table 4). Surprisingly, each of the revertants also harbored a second mutation within the *coq8* locus, indicating that the revertant phenotypes were perhaps due to intragenic suppression. Thus, in addition to the introduced A197V mutation, Rev-AL acquired the L237P mutation, Rev-BL acquired the P220S mutation, and Rev-CL, Rev-DL, and Rev-EL each acquired the same S232N mutation resulting from G695A (Table 4).

### Dominance tests reveal Rev-AL and Rev-BL are recessive and Rev-CL, Rev-DL, and Rev-EL are dominant

**Fig 3:**
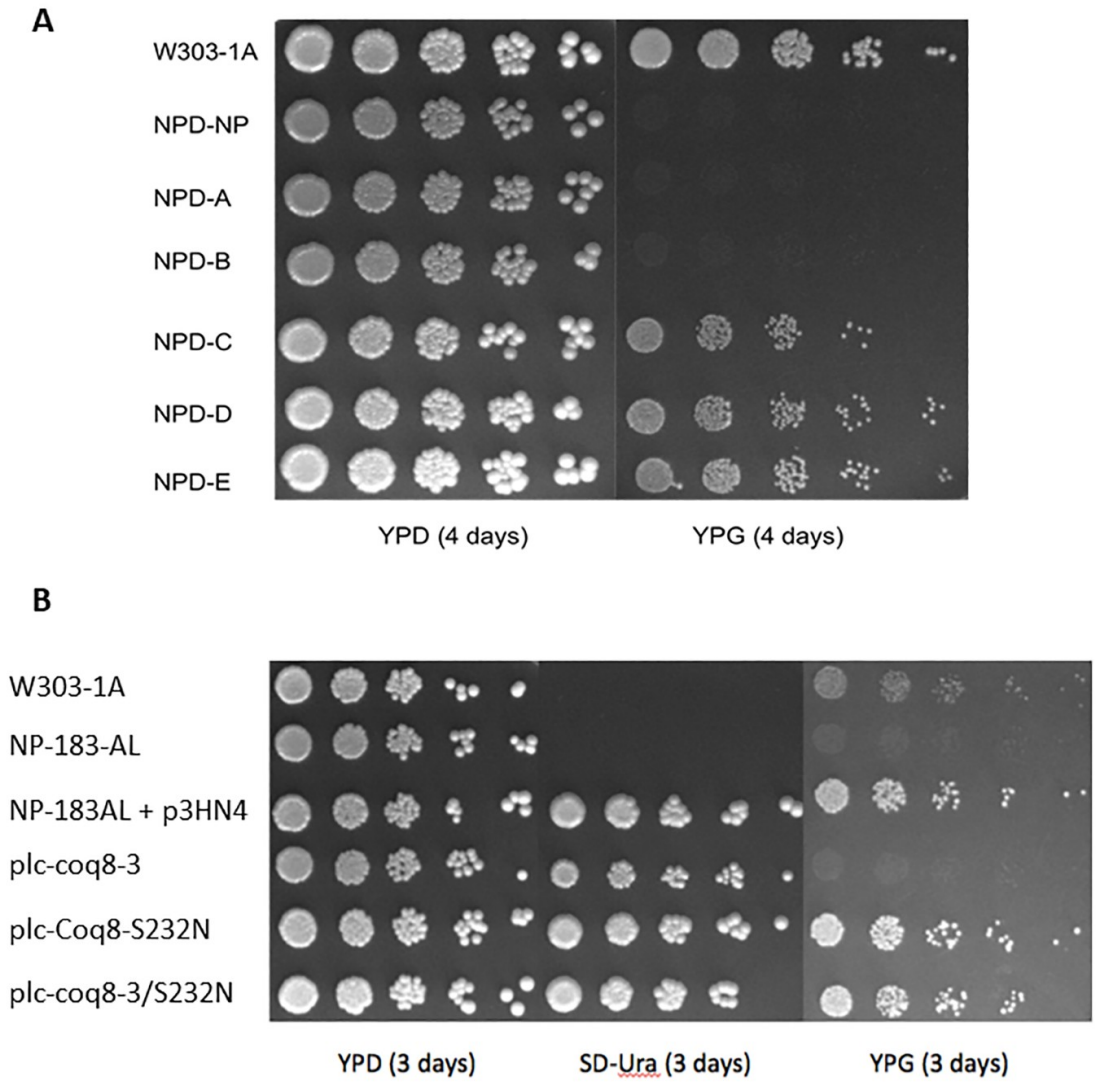
The Coq8-S232N substitution present in Rev-CL, Rev-DL, and Rev-EL is dominant, and its presence is sufficient to restore growth of the Coq8-A197V mutant on YPG. *A*, Each isolated revertant (Rev-AL, Rev-BL, Rev-CL, Rev-DL, and Rev-EL) was mated with parental mutant, NP-183BH and the respective diploid strains (NPD-A, NPD-B, NPD-C, NPD-D, and NPD-E) were isolated as described in *Materials and Methods*. As a control, NP-183AL was mated with NP-183BH to form a diploid strain NPD-NP containing two copies of the *coq8-3* mutation. The derived diploid strains were then plated on YPD, a fermentable carbon source, and YPG, a non-fermentable carbon source. The haploid wild-type strain, W303-1A, was also included. Plates were incubated at 30 °C for four days Panel *A* is representative of two independent biological replicate experiments (see S9 Fig). *B*, Expression of Coq8 harboring the dominant *SupRC* mutation present in Rev-CL rescues the growth of the NP183AL *coq8-3* mutant on non-fermentable carbon source medium. Yeast plate dilution assay was conducted on parental mutant, NP-183AL, transformed with the designated plasmids: plc-Coq8 (p3HN4), yeast low-copy *COQ8*; plc-A197V, yeast low-copy Coq8-A197V; plc-S232N, yeast low copy Coq8-S232N; or plc-A197V/S232N, yeast low copy Coq8-A197V/S232N. Each strain was cultured overnight in SD-Ura selective plate media, and the optical density (A_600nm_) adjusted to 0.2 with sterile PBS, and 2 μL of 5-fold serial dilutions were spotted onto each type of plate medium, as described in Fig 2. Cells were incubated at 30°C for three days. Panel *B* is representative of two independent biological replicate experiments (see S7 Fig).

To test dominance, each haploid revertant was mated with NP-183BH to create diploids (Table 1). The NPD-A and NPD-B diploids derived from Rev-AL and Rev-BL, respectively, were unable to grow on YPG, indicating that the L237P and P220S mutations were recessive suppressors (Fig 3A). Conversely, the NPD-C, NPD-D and NPD-E diploids each grew on YPG plate medium, and showed the S232N mutation was a dominant suppressor (Fig 3A).

To examine whether each of the revertant phenotypes could be attributed to a single nuclear mutation, the diploids were sporulated, and four to ten tetrads were dissected for each of the NPD-A, NPD-C, NPD-D and NPD-E diploids (S1–S3 Figs). In order to permit sporulation, the respiratory defective NPD-A and NPD-B diploids were first rescued by transformation with a low copy plasmid expressing wild-type *COQ8* (p3HN4, Table 2). Although this procedure enabled tetrad dissection of spores derived from NPD-A, we were not able to obtain tetrads from NPD-B. Four dissected spores from each tetrad were grown on YPD plate medium and replica-plated onto YPG plate medium. The haploid spores derived from NPD-A were first cured of p3HN4 prior to testing on YPG medium as described in *Materials and Methods*. Each of the diploid strains yielded tetrads that segregated 2:2 for respiration (S1–S3 Figs). These results indicated that the ability of Rev-AL, Rev-CL, Rev-DL and Rev-EL to respire was due to a single nuclear mutation.

### The S232N amino acid substitution is solely responsible for restoration of the growth of Rev-CL yeast on a non-fermentable carbon source

We wanted to determine whether the S232N change was responsible for the gain of function in the Rev-CL, Rev-DL and Rev-EL revertants expressing Coq8-A197V/S232N. To test this, p3HN4, a low copy plasmid carrying *COQ8*, was modified to create different variants. One variant possessed just the A197V mutation, a second had only the S232N mutation, and the final variant contained both point mutations (Table 4). The parental mutant, NP-183AL was transformed with each plasmid and tested for growth on a non-fermentable carbon source. As expected, NP-183AL with the plasmid expressing Coq8-A197V failed to grow on YPG plate medium (Fig 3B). However, significant growth was observed for NP-183AL containing the plasmid plc-Coq8-A197V/S232N and for the plasmid plc-Coq8-S232N (Fig 3B). This result confirmed that the S232N amino acid substitution was solely responsible for the gain of function of respiratory growth observed in the Rev-CL, Rev-DL and Rev-EL revertants. In subsequent studies, Rev-CL was used as the representative mutant for S232N.

### Structural prediction of yeast Coq8 reveals putative spatial organization of revertant mutations

**Fig 4:**
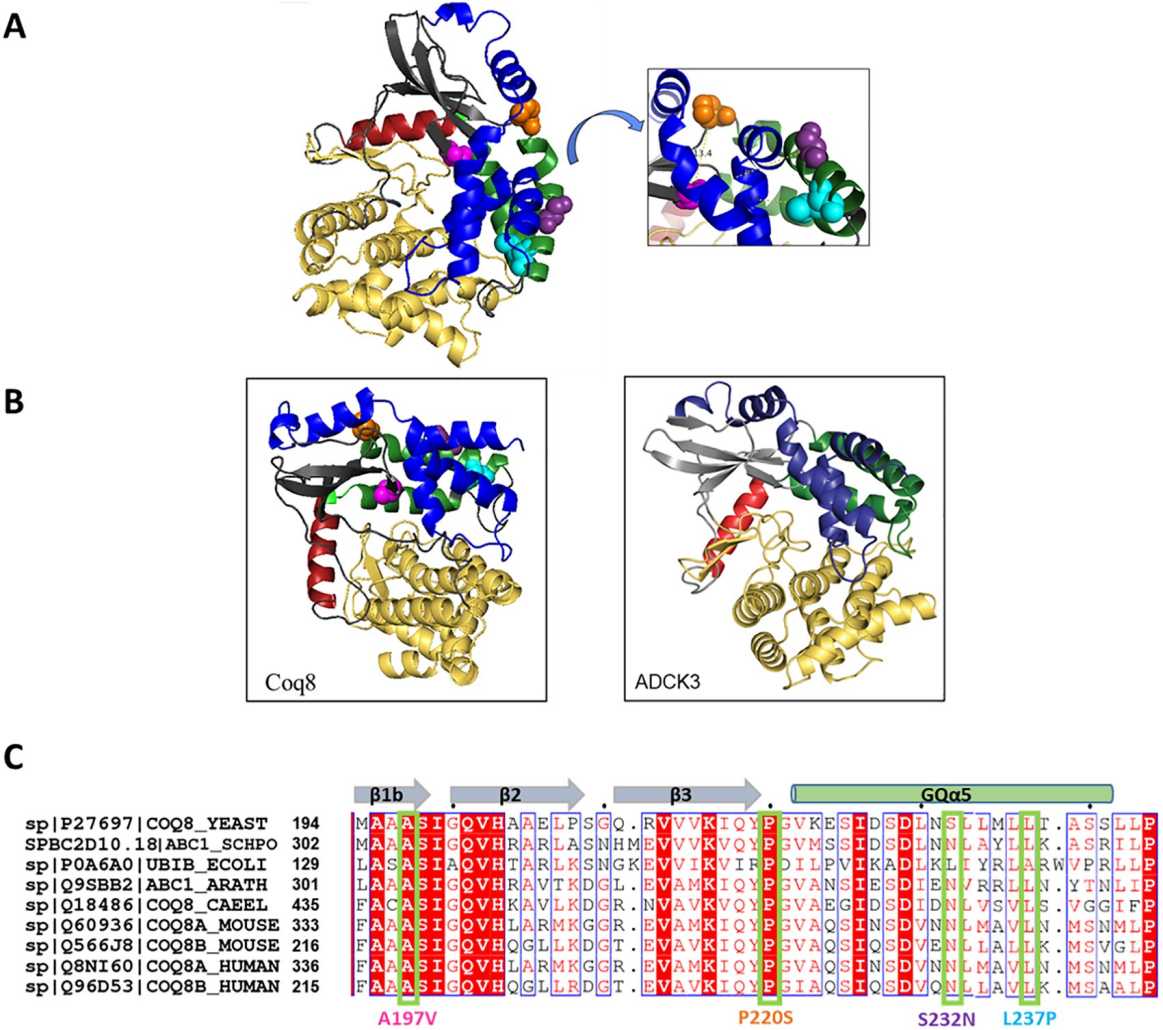
Structural prediction of *S*. *cerevisiae* Coq8 and sequence alignment with Coq8 homologs depict the sites of the A197V and suppressor mutations. *A*, PHYRE2 homology prediction of yeast Coq8, modeled to a 44% identity to PDB 4PED, corresponds to the partial structure of crystallized human COQ8A [13,41]. The structural features of Coq8 and COQ8A are color-coded as described previously [13]: The N-lobe folds into β-sheets, *gray* and a single helix αC, *red*; inserted between these features are the GQα5 and GQα6 helices, *green*; the C-lobe is shown in *yellow*; an N-terminal extension is shown in *blue*. The color-coding of the amino acids are: A197, *pink*; L237, *cyan*; P220, *orange*; and S232, *purple*. These locations are the sites of the parental mutant, Coq8-A197V and the three revertants, Rev-AL (L237P), Rev-BL (P220S), and Rev-CL (S232N). Rev-DL and Rev-EL also contained S232N. The predicted 32 amino acid mitochondrial targeting sequence of Coq8 [42] has been removed from the model to allow for the accurate depiction of the mature polypeptide. *A inset*, The predicted distances between the A197 and each of the amino acid substitutions present in Rev-AL, Rev-BL, and Rev-CL are shown on the structure following its rotation of 90°counterclockwise. *B*, Yeast Coq8 is depicted in the same orientation and color-coding as for the previously published ADCK3 [13]. *C*, Multiple sequence alignment and depiction of the locations of the mutations present in each of the yeast revertants. Secondary structure as predicted in the model of Coq8 is depicted above the yeast Coq8 sequence. A197V is present within the *coq8-3* parental mutant, and is present in each of the revertants. In addition, L237P occurs in Rev-AL, the P220S in Rev-BL, and the S232N is present in Rev-CL, Rev-DL and Rev-EL. The alignment included the designated Coq8 homologs from *S*. *cerevisiae*, *S*. *pombe*, *E*. *coli*, *A*. *thaliana*, *C*. *elegans*, *M*. *musculus*, and *H*. *sapiens*. The amino acid alignment was built using MUSCLE and visualized using EsPript [43].

A crystal structure for the yeast Coq8 polypeptide has not yet been reported. However a partial human COQ8A (ADCK3) structure was characterized and deposited as 4PED in the protein databank [13]. PHYRE2 homology modeling software was used to predict the putative structure of yeast Coq8, which was modeled at 44% identity to the partial human COQ8A structure 4PED (Fig 4A) [41]. Modeling yeast Coq8 in this manner relates the predicted locations of the suppressor mutations with respect to the previously published COQ8A domains. Yeast Coq8-A197 is the site of the A197V parental mutation, and is located in the Ala-rich loop of Protein Kinase-Like Motif I (PKLI), within the beta sheets that are part of the N-lobe (Fig 4; [13]). The suppressor mutations are all located next to, or within, the GQα5 motif, in what is termed the “N-lobe insert” (Fig 4; [13]). The relative predicted locations of L237 (L237P in Rev-AL), P220 (P220S in Rev-BL), and S232 (S232N in Rev-CL, Rev-DL and Rev-EL) are in close proximity to A197V in the primary sequence (Fig 4C). However, the mutations in Rev-AL and Rev-CL are estimated to reside 19 Å from the A197V mutation, and the P220S in Rev-BL is estimated to reside 13.4 Å from the A197V mutation (Fig 4A, inset). Moreover, it is apparent that L237, P220, and S232 residues are located on the surface of Coq8, remote from the predicted active site residues in the Ala-rich region of the PKL1 motif.

### Amino acid alignment indicates that S232N in Rev-CL is sustained as a conserved Asn in other eukaryotic Coq8 homologs

A multiple sequence alignment of yeast Coq8, *E*. *coli* UbiB, *A*. *thaliana* ABC1, *C*. *elegans* COQ-8, mouse COQ8A and COQ8B, and human COQ8A and COQ8B homologs shows the relative positions of the A197V and the three amino acid substitutions recovered in the revertants (Fig 4C). The mutations in Rev-AL (L237P) and in Rev-BL (P220S) both correspond to highly conserved residues in all eight Coq8 homologs. Intriguingly, with the exception of *S*. *cerevisiae*, all eukaryotic sequences examined in Fig 4C have a conserved Asn present at the relative position of yeast Coq8-S232. Since the Coq8-S232N substitution confers a dominant phenotype that over-rides the inactive Coq8-A197V mutation, it is possible that the Asn naturally present at this corresponding position in the other eukaryotic Coq8 homologs may confer an inherently more active state.

### Analyses of Q_6_ synthesis and content in the three revertant yeast strains as compared to wild-type yeast

**Fig 5:**
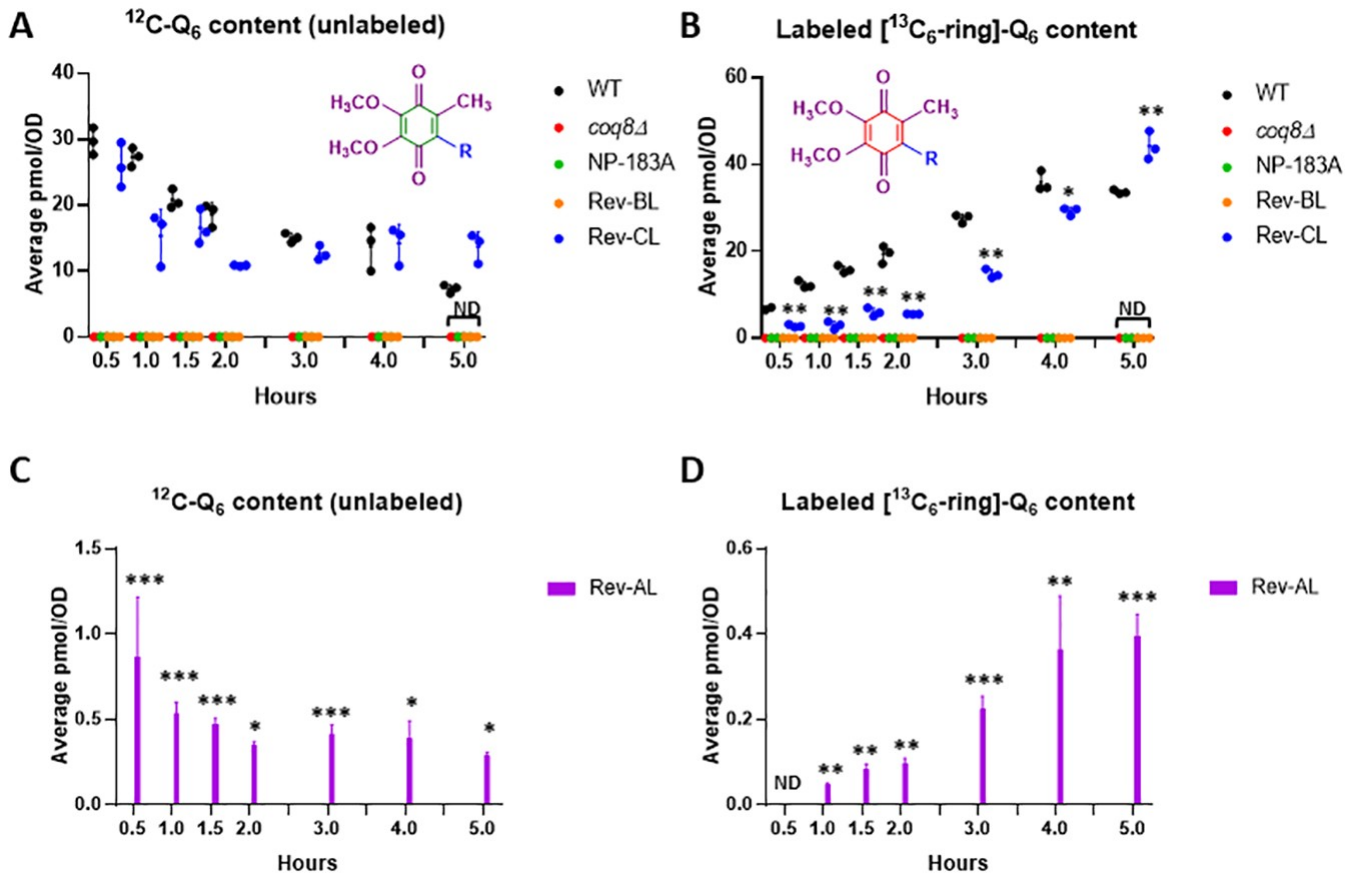
In YPD liquid medium, biosynthesis of Q_6_ in Rev-CL is nearly comparable to WT, while Rev-AL produces substantially lower levels of Q_6_ compared to WT, and in Rev-BL Q_6_ is not detected. *A*, Levels of unlabeled Q_6_ (^12^C-Q_6_) and *B*, *de novo* synthesized ^13^C_6_-Q_6_ (^13^C_6_-Q_6_) in each strain were determined at the designated time points after labeling with ^13^C_6_-4HB in YPD medium. Unlabeled Q_6_ and de novo labeled ^13^C_6_-Q_6_ were not detected in *coq8Δ*, NP-183A or in Rev-BL at any of the time points; this is denoted as “ND” at the 5 h time point for simplicity. *C* and *D*, The expanded scales show the levels of unlabeled and *de novo* ^13^C_6_-Q_6_ present in the Rev-AL strain. Error bars, S.D. of n = 3 biological replicates (unpaired Student’s t test between all strains compared to WT, with statistical significance represented by: *p<0.05, **p<0.005, ***p<0.0005). Results are shown for three independent biological replicate experiments.

Q_6_ *de novo* synthesis was measured following incubation with ^13^C_6_-4HB, a ring precursor of Q_6_ (Fig 1). In addition, total Q_6_ content, and Q_6_-intermediates were quantified in each of the six strains grown in YPD. Over the course of five hours, cells were harvested and lipid extracts were analyzed for unlabeled ^12^C-Q_6_ and *de novo* ^13^C_6_-Q_6_ levels using HPLC-tandem mass spectrometry. Rev-CL produced nearly comparable levels of unlabeled Q_6_ as WT, while producing slightly lower levels of *de novo* ^13^C_6_-Q_6_ (Fig 5A and 5B). The expanded scale presented in Fig 5C and 5D, shows that Rev-AL has significantly lower levels of unlabeled and *de novo* ^13^C_6_-Q_6_. Conversely, in these YPD cultures Rev-BL lacks detectable levels of unlabeled Q_6_ or labeled ^13^C_6_-Q_6_. In this regard it behaves identically to the Q-less *coq8Δ* and NP-183A mutants.

**Fig 6:**
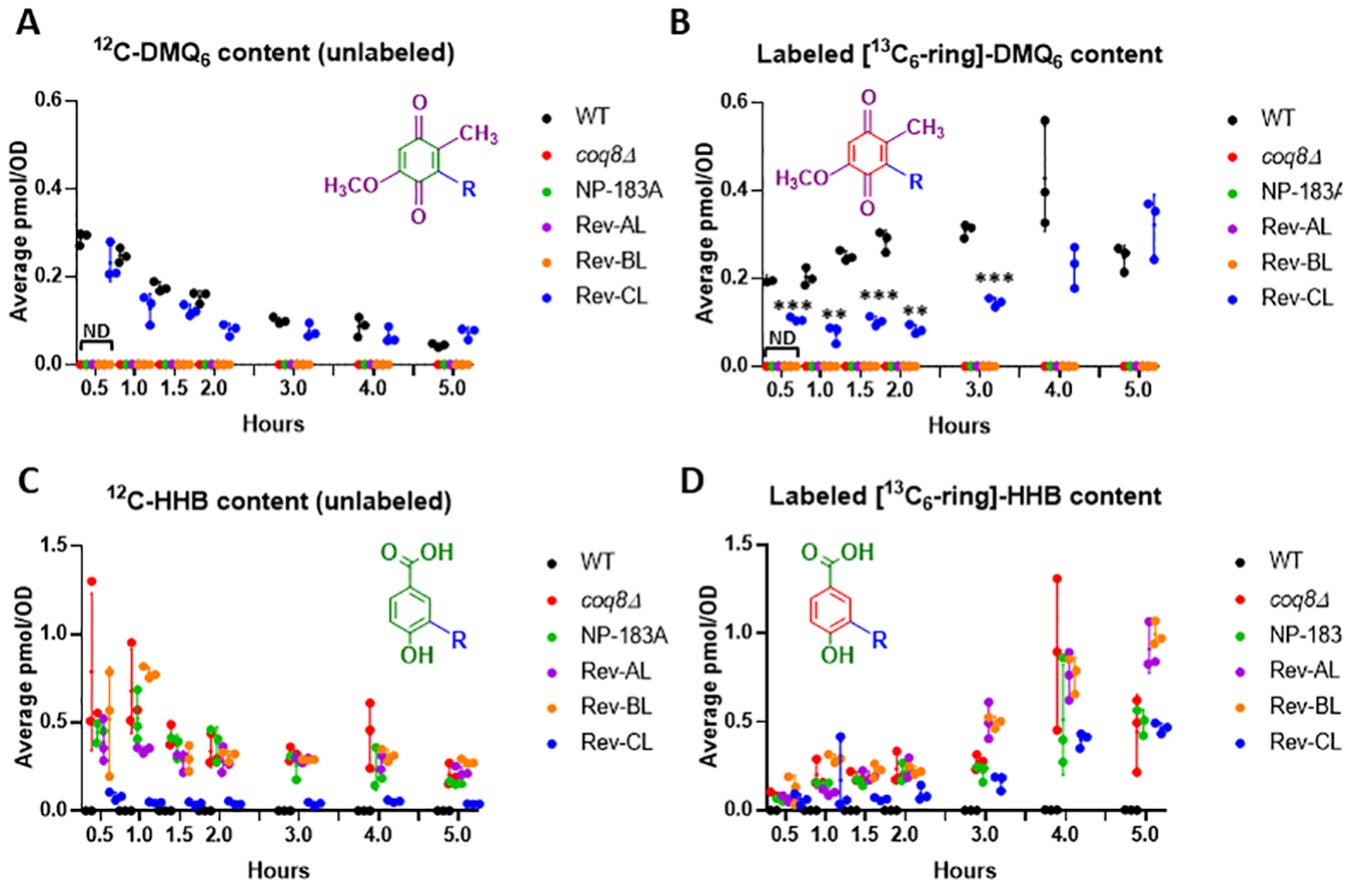
Rev-CL synthesizes late-stage intermediate DMQ_6_, while Rev-AL and Rev-BL accumulate the early-stage intermediate, HHB. *A*, Levels of unlabeled ^12^C-DMQ_6_; *B*, *de novo* synthesized ^13^C_6_-DMQ_6_; *C*, unlabeled ^12^C-HHB; and *D*, *de novo* synthesized ^13^C_6_-HHB were determined in WT, *coq8Δ*, NP-183A, Rev-AL, Rev-BL, and Rev-CL at the designated time points after labeling with ^13^C_6_-4HB in YPD medium. Error bars, S.D. of n = 3 biological replicates (unpaired Student’s t test between all strains compared to WT, with statistical significance represented by: *p<0.05, **p<0.005, ***p<0.0005). Panel A and B show non-detectable values for the lipids analyzed for *coq8Δ*, NP-183A, Rev AL, and Rev BL for all time points, indicated as “ND” for the first time point for simplicity. Panels *C* and *D* show non-detectable levels of HHB in WT. Results are shown for three independent biological replicate experiments.

WT and Rev-CL yeast strains that produced high levels of unlabeled Q_6_ and *de novo* ^13^C_6_-Q_6_, also contained comparable levels of unlabeled DMQ_6_ and *de novo* ^13^C_6_-DMQ_6_ (Fig 6A and 6B), the penultimate intermediate in Q_6_ biosynthesis (Fig 1). Rev-AL and Rev-BL, as well as the *coq8Δ* and NP-183A mutants produced substantially lower or undetectable levels of DMQ_6_ (Fig 6A and 6B). In contrast, these four mutant yeast strains contained relatively high levels of unlabeled HHB and ^13^C_6_-HHB as compared to WT (Fig 6C and 6D). HHB is an early intermediate in Q_6_ biosynthesis that frequently accumulates in yeast *coq*3 –*coq11* mutants with deficiencies in Q_6_ biosynthesis [9]. The relatively high accumulation of ^13^C_6_-HHB in Rev-CL suggests that *de novo* synthesis of ^13^C_6_-Q_6_ is impaired as compared to WT.

Overall, the lipid analyses in YPD medium over a five-hour time course show that the secondary mutation S232N in Rev-CL restored the capacity to produce Q_6_ to levels comparable to WT, while Rev-AL makes substantially lower levels of Q_6_. Surprisingly, Rev-BL failed to produce detectable amounts of unlabeled Q_6_ or labeled ^13^C_6_-Q_6_.

### Both Rev-CL and Rev-AL are able to synthesize Q_6_ in non-fermentable YPG medium

**Fig 7:**
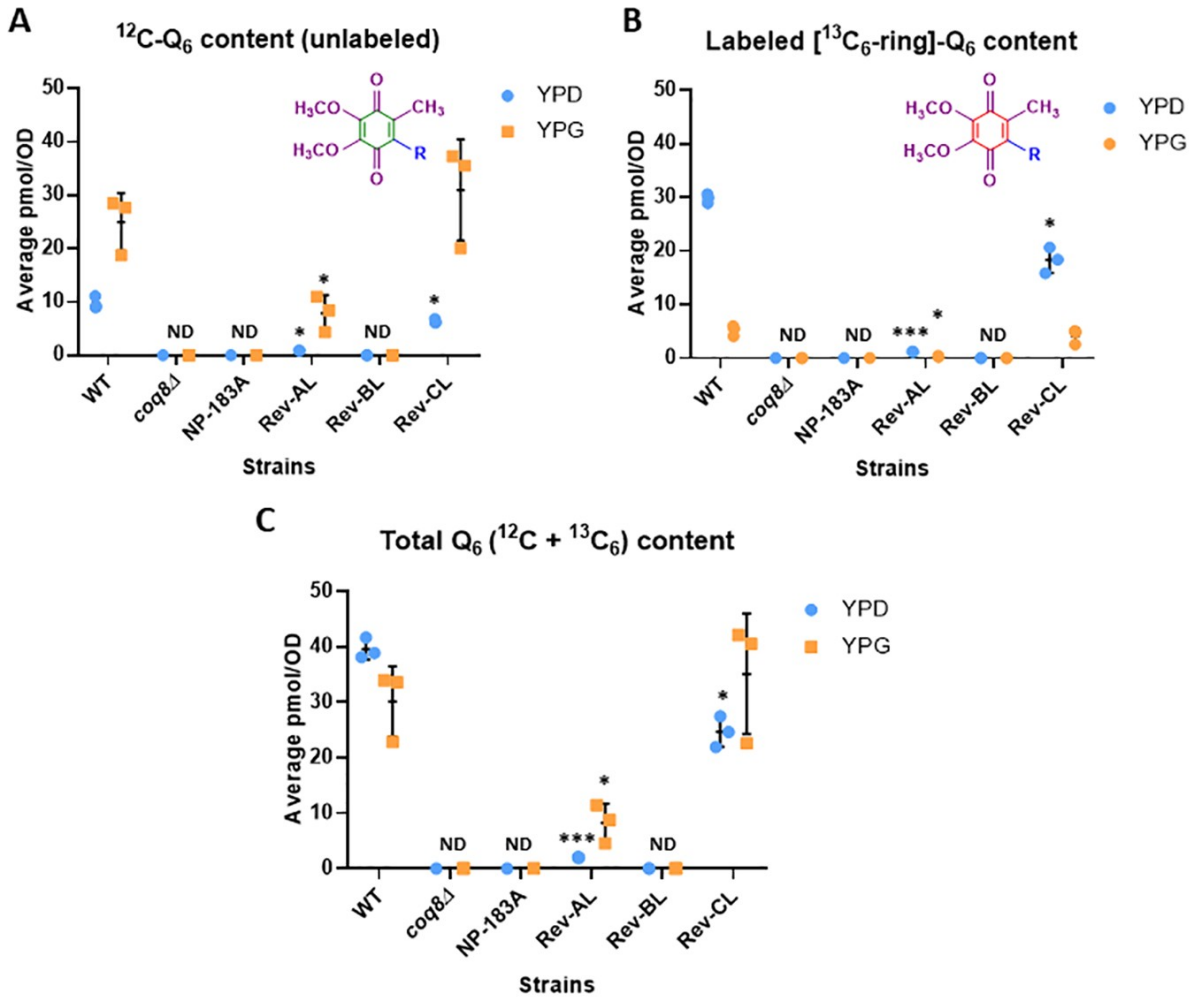
Rev-CL and Rev-AL are able to synthesize Q_6_ in YPG liquid medium, and Rev-CL produces comparable levels of total Q_6_ (^12^C-Q_6_ + ^13^C_6_-Q_6_) as WT. *A*, Levels of unlabeled Q_6_ (^12^C-Q_6_) and *B*, *de novo* synthesized ^13^C_6_-Q_6_ (^13^C_6_-Q_6_) in WT, *coq8Δ*, NP-183A, Rev AL, Rev BL, and Rev CL were determined after labeling with ^13^C_6_-4HB for 5 hours in YPD or YPG liquid medium. *C*, Total Q_6_ (^12^C-Q_6_ + ^13^C_6_-Q_6_) content is shown for all labeled strains in both YPD and YPG medium. Error bars, S.D. of n = 3 biological replicates (unpaired Student’s t test between all strains compared to WT in the same type of medium, with statistical significance represented by: *p<0.05, **p<0.005, ***p<0.0005). “ND” indicates lipids levels that were non-detectable in the indicated strains. Results are shown for three independent biological replicate experiments.

The lack of detectable Q_6_ in the YPD cultures of Rev-BL raised the question, how is this mutant able to grow successfully on YPG plates? In order to interrogate this further, each of the six strains was cultured in a YPG glycerol-based medium (non-fermentable) and in the YPD dextrose-based (fermentable) medium, and analyzed for unlabeled and *de novo* ^13^C_6_-Q_6_ production after five hours of incubation. Rev-CL exhibited comparable levels of unlabeled and *de novo* ^13^C_6_-Q_6_ to WT, in both types of medium (Fig 7A and 7B), and total (^12^C-Q_6_ +^13^C_6_-Q_6_) levels in Rev-CL were nearly identical to WT in YPG (Fig 7C). Rev-AL contained low amounts of unlabeled Q_6_, a trait that was more pronounced in YPG medium (Fig 7A). Curiously, neither unlabeled Q_6_ nor labeled ^13^C_6_-Q_6_ was detected in the Rev-BL yeast under these conditions (Fig 7).

### Rev-BL grown on solid YPG plate medium contains Q_6_

**Fig 8:**
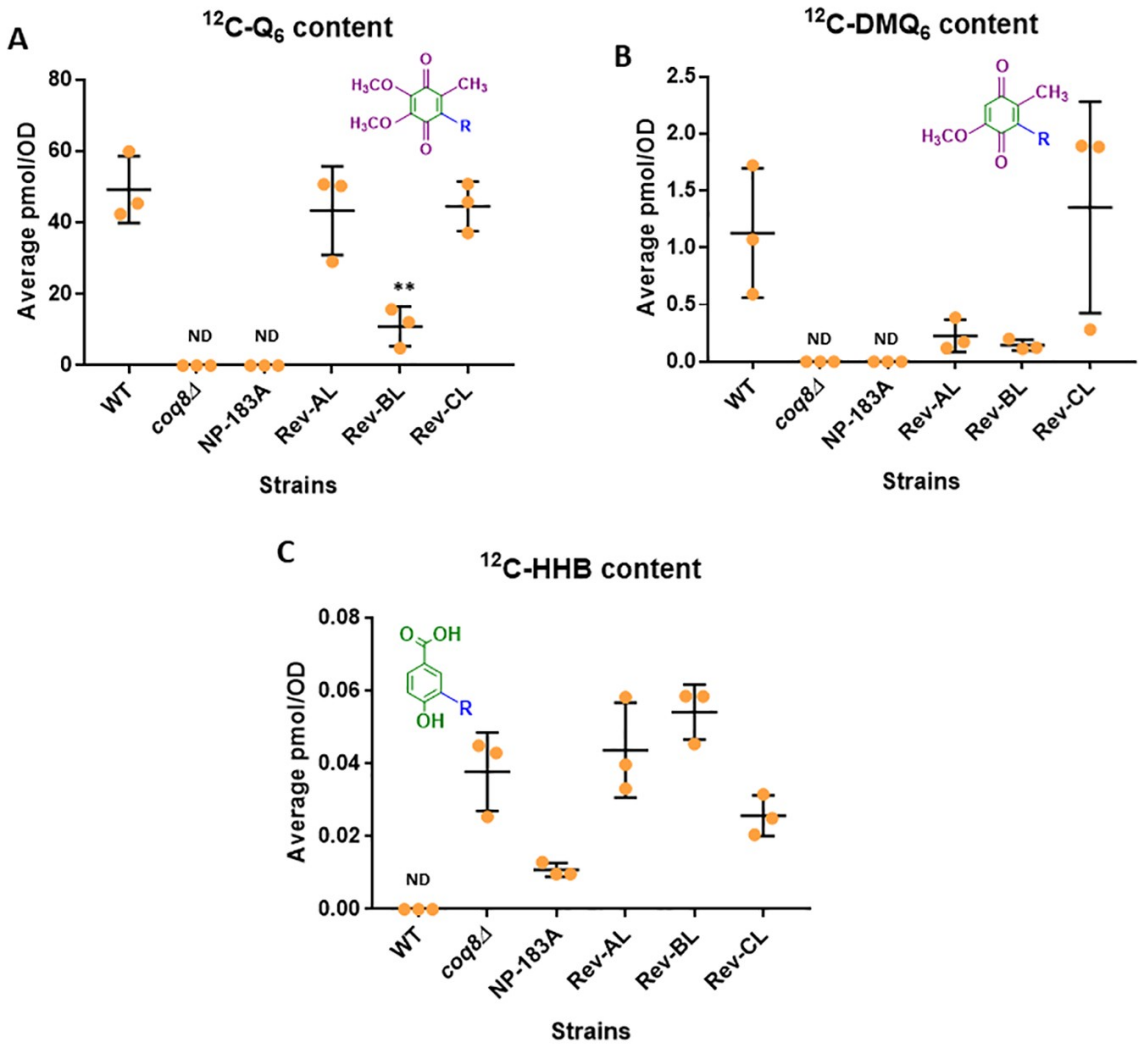
Assessment of Q_6_ and Q_6_-intermediates in strains following incubation on YPG plate medium reveals the presence of Q_6_ in Rev-BL. *A*, Levels of unlabeled ^12^C-CoQ_6_; *B*, ^12^C-DMQ_6_; and *C*, ^12^C-HHB in WT, *coq8Δ*, NP-183A, Rev AL, Rev BL, and Rev CL were determined on colonies that were cultured on YPG solid medium for two days. Error bars, S.D. of n = 3 biological replicates (unpaired Student’s t test between all strains compared to WT, with statistical significance set at p<0.05). “ND” indicates lipids levels that were undetectable in the indicated strains. Results are shown for three independent biological replicate experiments.

Since culture for five hours in liquid YPG medium failed to allow Rev-BL to produce any detectable amounts of Q_6_, we recovered yeast from colonies that were cultured on YPG plate medium. Under these growth conditions, WT, Rev-AL, Rev-BL and Rev-CL showed growth (Fig 2) and successful production of Q_6_ (Fig 8A). In fact, WT, Rev-CL, and Rev-AL showed comparable levels of Q_6_, while Rev-BL produced lower amounts of Q_6_ (Fig 8A). These results show that under conditions of growth on YPG plate medium, Rev-BL is able to produce Q_6_. It has been shown that growth on YPG plate medium requires only 0.2 to 3% of the “baseline” levels of Q_6_ [9,11,44,45]. This may explain the low but comparable growth of Rev-BL and Rev-AL observed on YPG plate medium (Fig 2B). As controls, the *coq8Δ* and NP-183A yeast strains were also applied to the YPG plate medium. Although these yeast fail to grow on YPG medium, the region of the plate containing the applied yeast cells was recovered, and analyzed for Q_6_ and Q_6_-intermediates as described in *Materials and Methods*. Neither Q_6_ nor DMQ_6_ were detected in these lipid extracts, although an early Q_6_-intermediate HHB, was detected (Fig 8).

### Growth in nutrient conditions that facilitate the study of mitochondrial function reveals the capacity of all three revertants to produce Q_6_

**Fig 9:**
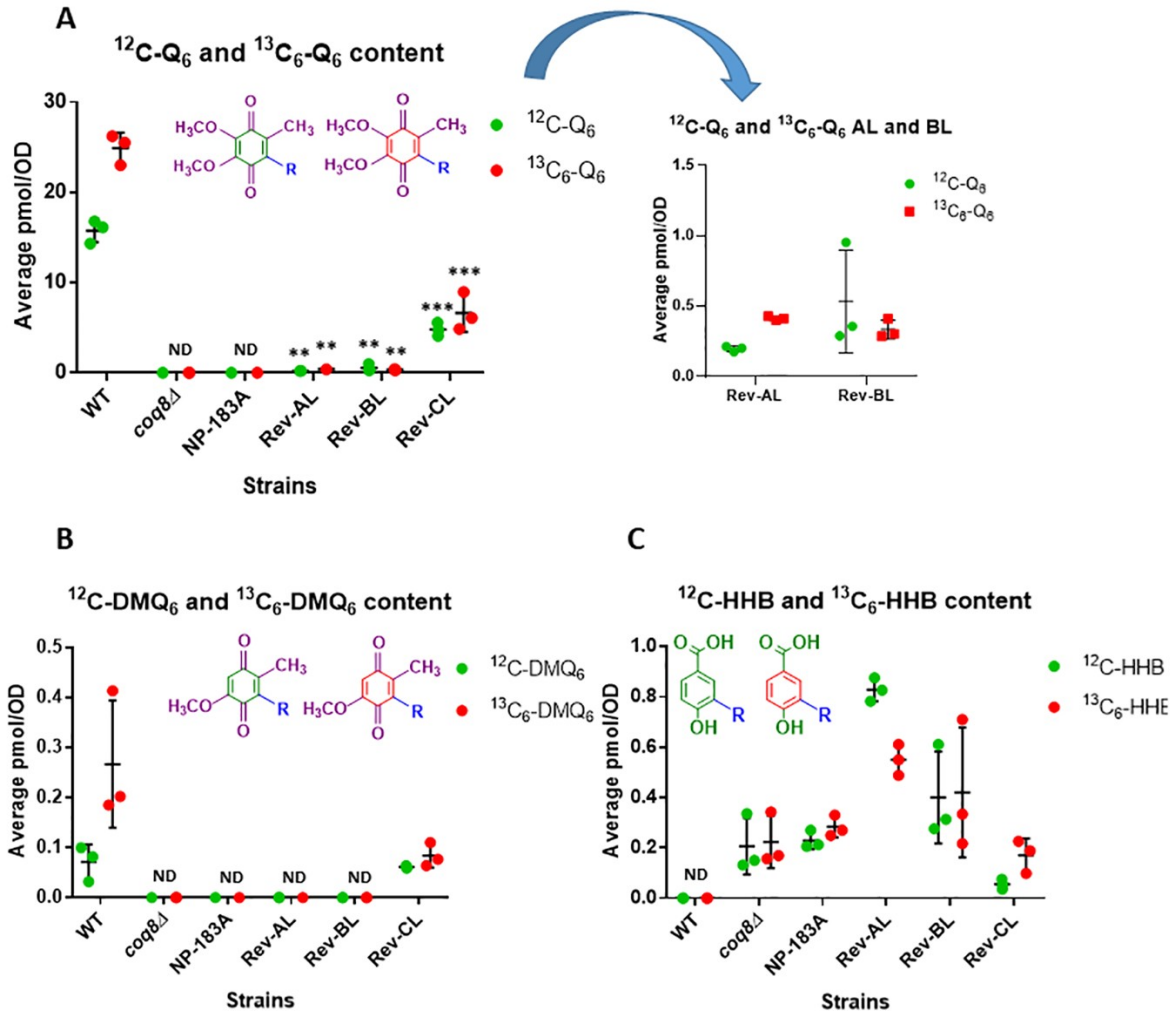
Growth in YPGal (a nonrepressive carbon source) reveals all three revertants are capable of *de novo* ^13^C_6_-Q_6_ production. *A*, Levels of unlabeled ^12^C-Q_6_ and *de novo* ^13^C_6_-Q_6_; *B*, unlabeled ^12^C-DMQ_6_ and *de novo* ^13^C_6_-DMQ_6_; *C*, unlabeled ^12^C-HHB and *de novo* ^13^C_6_-HHB in WT, *coq8Δ*, NP-183A, Rev-AL, Rev-BL, and Rev-CL were determined in cultures of yeast labeled for 5 hours with ^13^C_6_-4HB in YPGal + 0.1% Dextrose liquid media. The expanded y axis in the panel *A* inset demonstrates the levels of Q_6_ production in Rev-AL and Rev-BL. Error bars, S.D. of n = 3 biological replicates (unpaired Student’s t test between all strains compared to WT, with statistical significance represented by: *p<0.05, **p<0.005, ***p<0.0005). “ND” indicates lipids levels that were non-detectable and lower than background in the indicated strains. Results are shown for three independent biological replicate experiments.

In order to study mitochondrial function in yeast strains, rich growth medium containing 2% galactose (YPGal) is often used as a non-repressing carbon source [46]. The use of this growth medium circumvents the repression of mitochondrial function mediated by dextrose. For this analysis, a predominantly galactose carbon source was used (YPGal with 0.1% dextrose). Under this condition, Rev-CL showed a decreased capacity to produce both unlabeled and *de novo* ^13^C_6_-Q_6_, as compared to WT (Fig 9A), while Rev-AL and Rev-BL produced substantially lower amounts of unlabeled and ^13^C_6_-Q_6_ than Rev-CL (Fig 9A, inset). Indeed, only WT and Rev CL showed detectable levels of DMQ_6_, while all the strains except WT showed accumulation of the early precursor, HHB (Fig 9B and 9C).

### The suppressor mutations in Rev-AL, Rev-BL, and Rev-CL result in different content of the CoQ synthome polypeptides

**Fig 10:**
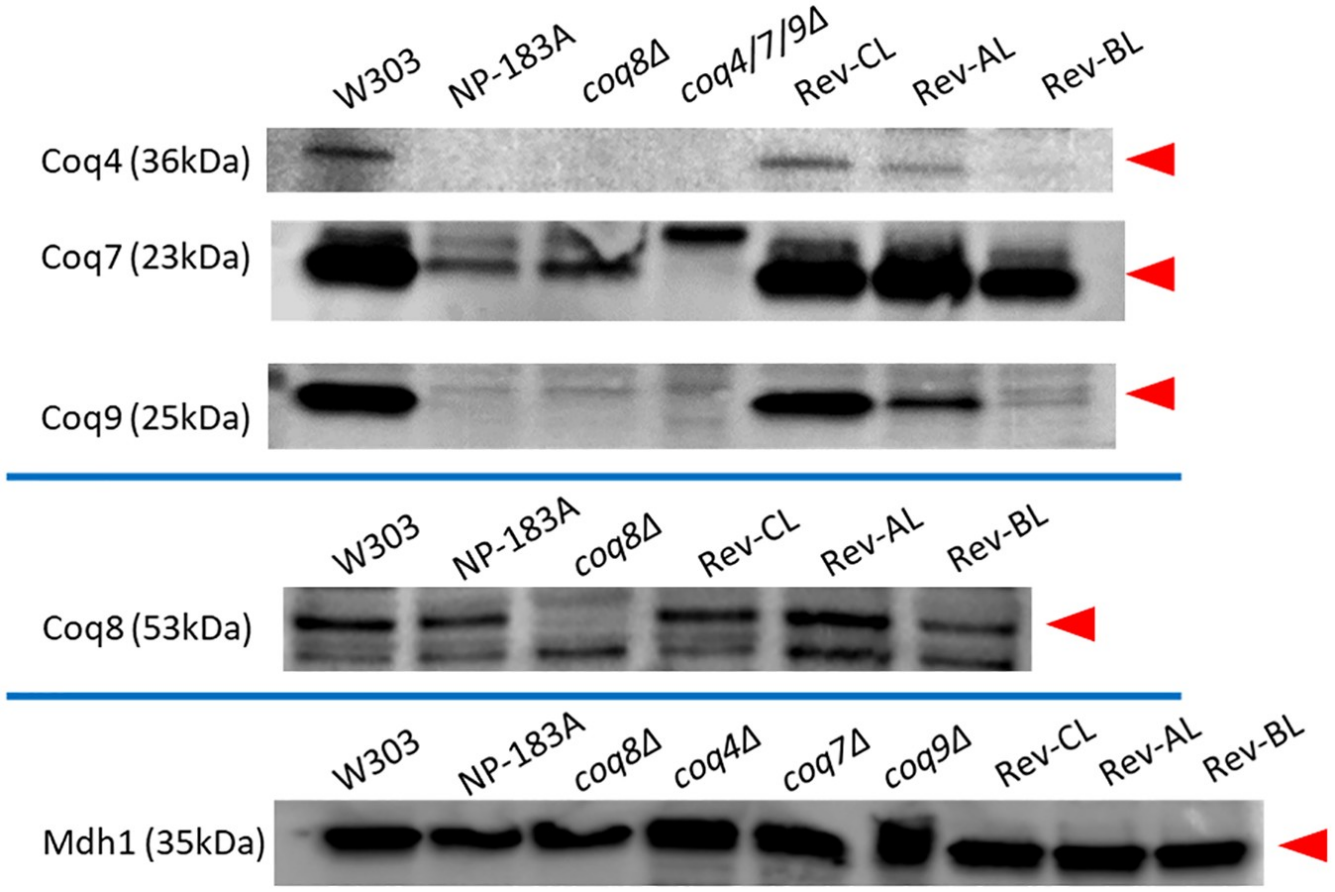
Amounts of indicator Coq polypeptides in mitochondria isolated from Rev-CL are restored to near wild-type content. The amounts of Coq4, Coq7, Coq8, and Coq9 polypeptides were determined by SDS-PAGE and immunoblotting. Samples were separated on 12% SDS-PAGE gels and then transferred to PVDF membranes for immunoblotting with antisera to the designated yeast Coq polypeptides. 25 μg of purified mitochondria was analyzed for each strain. Arrows indicate each antibody-detected protein in their respective blots; a *coq* null strain in each respective Coq polypeptide blot was used as a reference for the absence of the desired band of interest. Blots were performed at least two times each (see S4–S6 Figs). The Mdh1 blot serves to validate the samples used in all the blots; the samples that were loaded in the Mdh1 blot are those of the same preparation as used for the Coq4, Coq7, Coq8, and Coq9 blots.

We sought to investigate the effects of the Coq8 suppressors on the content of sensitive indicator Coq polypeptides. These indicator polypeptides include Coq4, Coq7, and Coq9, because they serve key roles in maintaining the high molecular mass CoQ synthome [12]. In order to track these indicator Coq polypeptides, mitochondria were subjected to immunoblotting with the primary antibodies described in Table 3. Interestingly, Rev-CL contained nearly wild-type content of the Coq4 and Coq9 polypeptides, while Rev-AL and particularly Rev-BL had decreased amounts of Coq4 and Coq9 (Fig 10). This is quite intriguing, since Coq4 has previously been shown to serve as a central organizer of the CoQ synthome [12]. The content of Coq7 is retained at near wild-type content in Rev-CL and Rev-AL, but was slightly decreased in Rev-BL. This is consistent with the higher content of Q_6_ in Rev-CL, since Q_6_ production relies on Coq4, Coq7, and Coq9 [9]. It appears as though the Rev-AL and Rev-CL exhibit a near normal amount of Coq8 polypeptide as compared to WT, while the content of Coq8 polypeptide is lower in Rev-BL mitochondria (Fig 10).

## Discussion

Coq8 is a member of an ancient family of atypical protein kinases with essential roles in Q_6_ biosynthesis. Coq8 facilitates the assembly of the CoQ synthome, a multisubunit complex that is essential for the biosynthesis of Q_6_ in yeast [12]. Coq8 also mediates the organization of the CoQ synthome into discrete domains within mitochondria, observed as puncta located adjacent to ER-mitochondria contact sites [23]. These functions are conserved in COQ8A, the human ortholog of yeast Coq8 [23]. *E*. *coli* UbiB, yeast Coq8 and human COQ8A possess ATPase activity that is stimulated by analogs of Q-intermediates [21], although the mechanism(s) by which Coq8 or COQ8A mediate the assembly or spatial organization of the CoQ synthome (or Complex Q) is still mysterious [23,47].

In this study we used a yeast Coq8-A197V mutant previously characterized as Q-less and respiratory deficient [15]. The A197V mutation occurs within the crucial Ala-rich PKL-motif I of yeast Coq8, that replaces the Gly-rich nucleotide-binding loop normally present in canonical protein kinases [13] (Fig 4C). Thus, the A197V mutation occurs within the presumed active site of the Coq8 polypeptide, and is likely to impact the binding of ATP and its hydrolysis. Here, we characterized spontaneous revertants of the Coq8-A197V mutant that were isolated following long-term culture in growth medium containing glycerol, a non-fermentable carbon source. Each revertant (Rev-AL, Rev-BL, Rev-CL, Rev-DL and Rev-EL) acquired the ability to grow on medium containing glycerol and to synthesize Q_6_. Surprisingly, each revertant was found to contain a secondary mutation within the *COQ8* gene, suggesting that the recovery of glycerol growth and Q_6_ synthesis resulted from an intragenic suppressor mutation.

**Fig 11:**
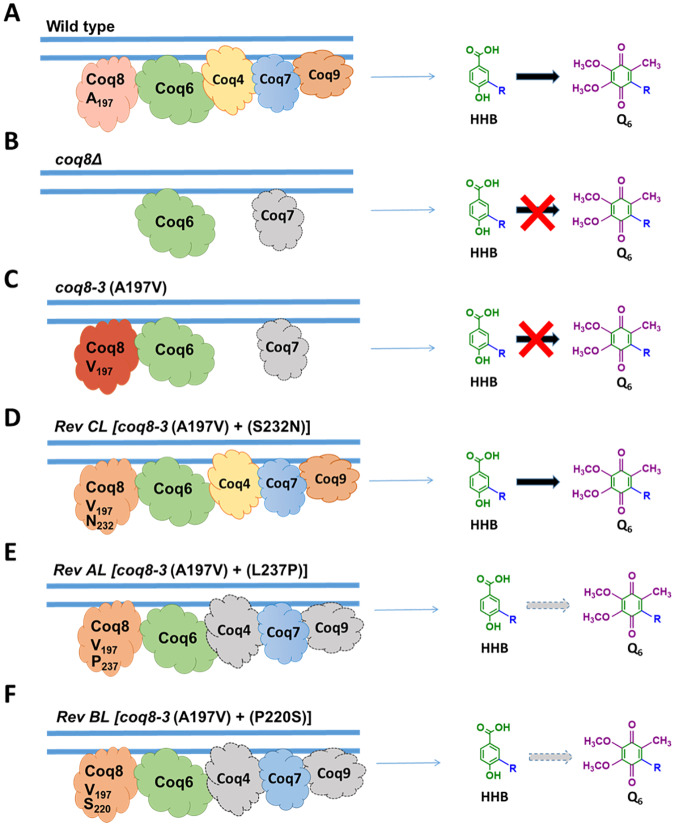
Model for the restoration of Q_6_ biosynthesis in three revertants. *A*, Under conditions of Coq8 expression in wild-type yeast, benchmark values of Q_6_ production are noted and the wild-type amounts of key Coq polypeptides (Coq4, Coq7, Coq8, and Coq9) are defined. *B*, the yeast *coq8*Δ mutant lacks Q_6_, has a lower content of the Coq7 polypeptide, the Coq4, Coq8, and Coq9 polypeptides are not detectable, and the early intermediate HHB accumulates. *C*, the yeast mutant expressing the Coq8-A197V polypeptide lacks Q_6_, has a lower content of the Coq7 polypeptide, the Coq4 and Coq9 polypeptides are not detectable, and the early intermediate HHB accumulates. *D*, The dominant Rev-CL revertant restores Q_6_ production and the stability of the Coq4, Coq7 and Coq9 polypeptides. *E*, The recessive Rev-AL revertant has partially restored Q_6_ biosynthesis. However, amounts of the Coq4 and Coq9 polypeptides are low and this results in greatly impaired synthesis of Q_6_. *F*, The recessive Rev-BL revertant exhibits very low levels of Q_6_ biosynthesis, and has dramatically decreased content of the Coq4 and Coq9 polypeptides, even though Coq8 and Coq7 polypeptides remain readily detectable.

Each of the intragenic suppressor mutations was determined to reside next to or within the GQα5 helix of Coq8. The GQα5 helix is one of two helices that form an insert located between the beta sheet (β3) and the αC helix (Fig 4). This insert is part of a distinct and conserved feature present in each of the PKL members of the UbiB family [13]. Each intragenic suppressor mutation was predicted to lie near the surface of Coq8, and to range a distance of 14–19 Å from the A197V mutation (Fig 4). It is tempting to speculate that the surface location of the Coq8 suppressor mutations allows for interactions of Coq8 with other Coq subunit proteins of the CoQ synthome. Because expression of Coq8-A197V/S232N is able to dominantly suppress the parental mutant phenotype, it may serve to displace the Coq8-A197V polypeptide. In contrast the recessive intragenic suppressors are unlikely to be able to displace the Coq8-A197V polypeptide (Fig 11).

Differences between the two classes of revertants (dominant and recessive) are also evident from their capacity to synthesize Q_6_. Rev-CL displayed the capacity to synthesize quantities of Q_6_ comparable to WT yeast. Rev-AL and Rev-BL, however, produced much lower amounts of Q_6_, yet still showed the ability to make significant quantities, when cultured on YPGal + 0.1% dextrose liquid medium or when cultured on YPG plate medium. Thus, we hypothesized that the Coq8-A197/S232N polypeptide present in Rev-CL may act to restore the amouts of Coq polypeptides that are components of the CoQ synthome.

Determining the levels of sensitive indicator Coq polypeptides tested this idea. Rev-CL displayed robust amounts of Coq4, Coq7, and Coq9 polypeptides. The Coq8-A197V/S232N polypeptide was also present at amounts comparable to Coq8 in WT. This is consistent with the high Q_6_ synthesis in the Rev-CL yeast. In contrast, the recessive revertants, Rev-Al and Rev-BL produced much less Q_6_, and retained low amounts of the Coq4 and Coq9 indicator polypeptides.

It is notable that the Coq8-A197V/S232N polypeptide in Rev-CL allows for efficient respiration and a near perfect restoration of Q_6_ production. In eukaryotic Coq8 homologs, an Asn residue normally occupies this same location as S232 (Fig 4C). It is possible that the yeast Coq8-S232N point mutation restores Q_6_ biosynthesis by mimicking the sequence of higher order eukaryotic Coq8 orthologs.

A distinct mutation of A197G in Coq8 has been shown to decrease Q_6_ content and inhibit growth on glycerol containing medium [13], and also results in enhanced ATPase activity and cis-autophosphorylation [21,22]. Similar trends of ATPase and cis-autophosphorylation are noted for the A339G mutation in human COQ8A, which corresponds to Coq8-A197G [21,22]. It would be interesting to perform similar activity assays on the Coq8-A197V and each of the three revertants.

Protein kinase activity in trans has not been demonstrated for any of the UbiB, Coq8, or COQ8A PKL orthologs [22]. It is possible that the full-length activity of Coq8 may show different properties than the truncated versions used in activity assays to date [48]. A recent report identifies a PKL family member with distinct ATP-dependent ligation activity [49]. There is precedent in *A*. *thaliana* for trans phosphorylation mediated by an ABC1K homolog of the UbiB family. ABC1K1 has been shown to phosphorylate VTE1, a plastoglobule protein involved in vitamin E synthesis [50]. ABC1K1 is one of five ABC1K homologs located within the plastoglobule, a lipid droplet within the plastid that contains prenylated lipids including plastoquinone and vitamin E [51]. Finally, it is possible that phosphorylation sites may control transport into the mitochondria, since several sites present in Coq7 and Coq9 polypeptides are located near the amino terminus, and are not present in the full length polypeptide [15,52,53]. The phosphatase enzymes Ptc7 in yeast and PPTC7 in human cells have recently been shown to aid in import of mitochondrial proteins [54]. It will be important to determine the effect of the revertant mutations on the role of full-length Coq8 in order to understand its mechanisms of action in Q biosynthesis.

## Supporting information

S1 Fig Sporulation of NPD-C diploid yeast with tetrad dissection and test for 2:2 segregation of growth on YPG plate medium. *A*, Tetrad dissection is shown for six sets of four spores (rows B-F and H); *B*, YPG growth tested for each of the colonies in row B (B1-B4); *C*, YPG growth tested for each of the colonies in row C (C1-C4). The 2:2 segregation of growth on YPG plate medium shown is representative of twelve sets of tetrads generated from the NPD-C diploid yeast strain.
(PDF)

S2 Fig Sporulation of NPD-E diploid yeast with tetrad dissection and test for 2:2 segregation of growth on YPG plate medium. *A*, Tetrad dissection is shown for five sets of tetrads containing four spores (rows A-E); *B*, YPG growth tested for each of the colonies in rows A (A1-A4) and B (B1-B4); *C*, YPG growth tested for each of the colonies in rows C (C1-C4) and D (D1-D4). The 2:2 segregation of growth on YPG plate medium shown is representative of five sets of tetrads generated from the NPD-E diploid yeast strain.
(PDF)

S3 Fig Test of four tetrads obtained from NPD-A diploid yeast for 2:2 segregation of growth on YPG plate medium. The 2:2 segregation of growth on YPG plate medium is shown for four sets of tetrads generated from the NPD-E diploid yeast strain. *A*-*D* show YPG growth tested for each of the colonies obtained from four independent tetrad dissections obtained from the sporulation of NPD-A diploid yeast strain. Also shown is the YPG positive growth of the W303-1A WT and the lack of YPG growth of the NP-183AL parental haploid strain.
(PDF)

S4 Fig Unedited and uncropped full image western blots, Round 1. Full western blot images for Coq4, Coq7, Coq8, Coq9, and Mdh1 completed for all strains with samples of purified mitochondria. These westerns are the first of two replicates completed. The order for each lane are: ladder, WT, NP-183A, *coq8Δ*, *coq4/7/9Δ*, Rev-CL, Rev-AL, Rev-BL. Only for the Coq8 blot is the order: ladder, WT, NP-183A, *coq8Δ*, Rev-CL, Rev-AL, Rev-BL. The ladder molecular weight is labeled for the region flanking the respective band, which is annotated by a red arrow.
(PDF)

S5 Fig Unedited and uncropped full image western blots, Round 2 and used in Fig 10. Full western blot images for Coq4, Coq7, Coq8, Coq9, and Mdh1 completed for all strains with samples of purified mitochondria. These westerns are the second of two replicates completed and were used to prepare Fig 10. The order for each lane are: ladder, WT, NP-183A, *coq8Δ*, *coq4/7/9Δ*, Rev-CL, Rev-AL, Rev-BL. Only for the Coq8 blot is the order: ladder, WT, NP-183A, *coq8Δ*, Rev-CL, Rev-AL, Rev-BL. The Mdh1 blot contains all the samples used for the blots, to serve as a control. The ladder molecular weight is labeled for the region flanking the respective band, which is annotated by a red arrow.
(PDF)

S6 Fig Coq4 blot validated to affirm results of the respective blot from S5 Fig. The full image of an additional round of the Coq4 blot are shown to validate the results of S5 Fig. This is because Coq4 showed different results between S4 and S5 Figs. The results of S5 Fig appear to reflect the most consistent trends of the Coq4 polypeptide amongst all the strains. These full westerns are unedited and uncropped. The order for each lane are: ladder, WT, NP-183A, *coq8Δ*, *coq4/7/9Δ*, Rev-CL, Rev-AL, Rev-BL. Note that the background species are not uniform between the lanes for each strain, probably due to the polyclonal sera used in these experiments.
(PDF)

S7 Fig Validation of experiment of Fig 3B, phenotype rescue as a result of plasmid expression. Second repetition of the phenotype rescue experiment contained in Fig 3B.
(PDF)

S8 Fig Validation of experiment of Fig 2, plate dilution assay and growth on YPD and YPG plate medium. Second repetition of the plate dilution assessment of growth present in Fig 2.
(PDF)

S9 Fig Validation of of experiment in Fig 3A, plate dilution assay and growth on YPD and YPG plate medium of diploid strains. Second repetition of the plate dilution assessment of growth presented in Fig 3A.
(PDF)

S1 Raw images (PDF)

S1 File (ZIP)
