# Supplementary material for: Intragenic suppressor mutations of the COQ8 protein kinase homolog restore coenzyme Q biosynthesis and function in Saccharomyces cerevisiae
Source: PLoS One. 2020 Jun 1;15(6):e0234192. doi: 10.1371/journal.pone.0234192 (PMC7263595; doi:10.1371/journal.pone.0234192)

S1 Fig. Sporulation of NPD-C diploid yeast with tetrad dissection and test for 2:2 segregation of growth on YPG plate medium

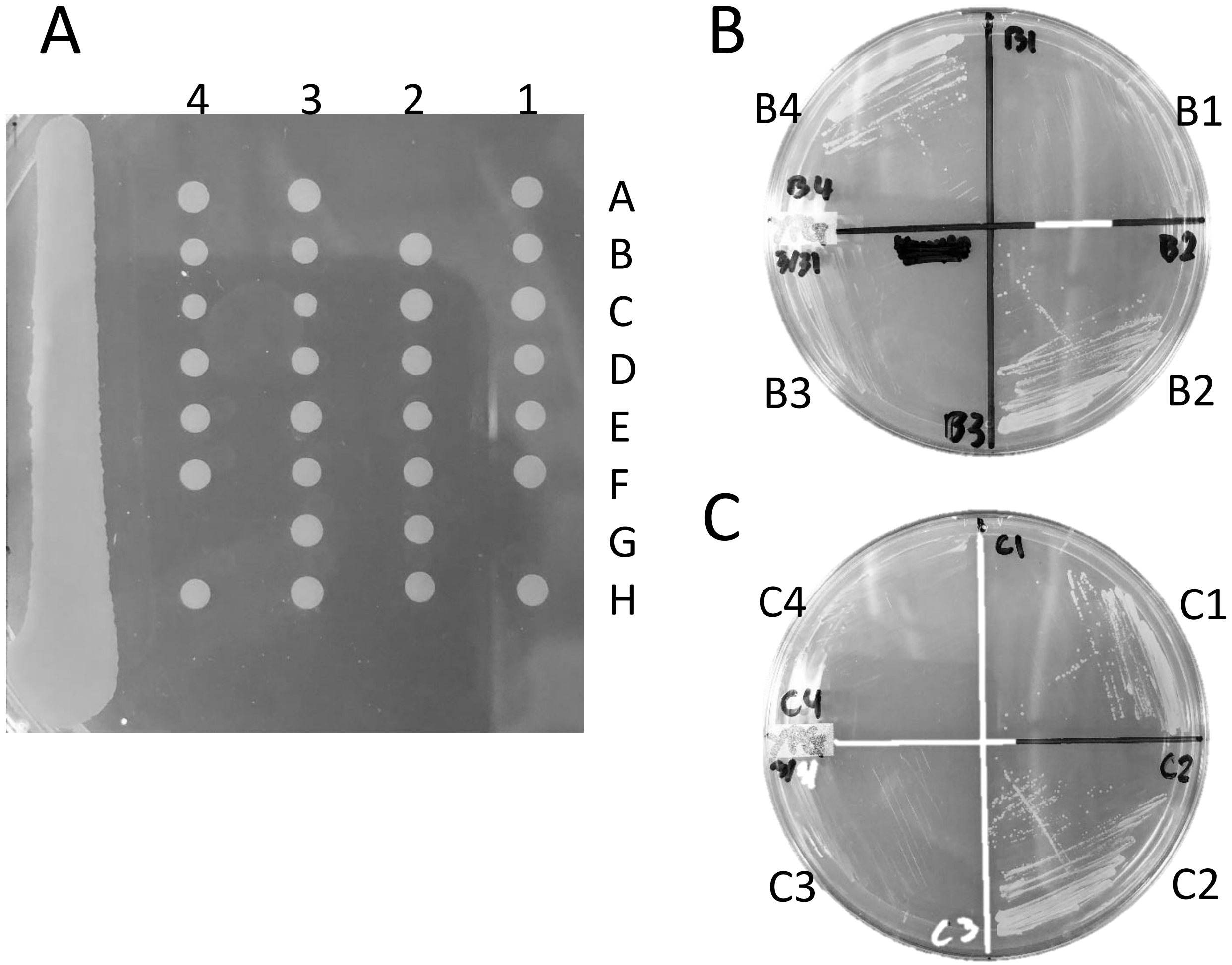

Supplement: S1 Fig — A, Tetrad dissection is shown for six sets of four spores (rows B-F and H); B, YPG growth tested for each of the colonies in row B (B1-B4); C, YPG growth tested for each of the colonies in row C (C1-C4). The 2:2 segregation of growth on YPG plate medium shown is representative of twelve sets of tetrads generated from the NPD-C diploid yeast strain. (PDF) [file pone.0234192.s001.pdf]
