# Supplementary material for: Intragenic suppressor mutations of the COQ8 protein kinase homolog restore coenzyme Q biosynthesis and function in Saccharomyces cerevisiae
Source: PLoS One. 2020 Jun 1;15(6):e0234192. doi: 10.1371/journal.pone.0234192 (PMC7263595; doi:10.1371/journal.pone.0234192)

S2 Fig. Sporulation of NPD-E diploid yeast with tetrad dissection and test for 2:2 segregation of growth on YPG plate medium

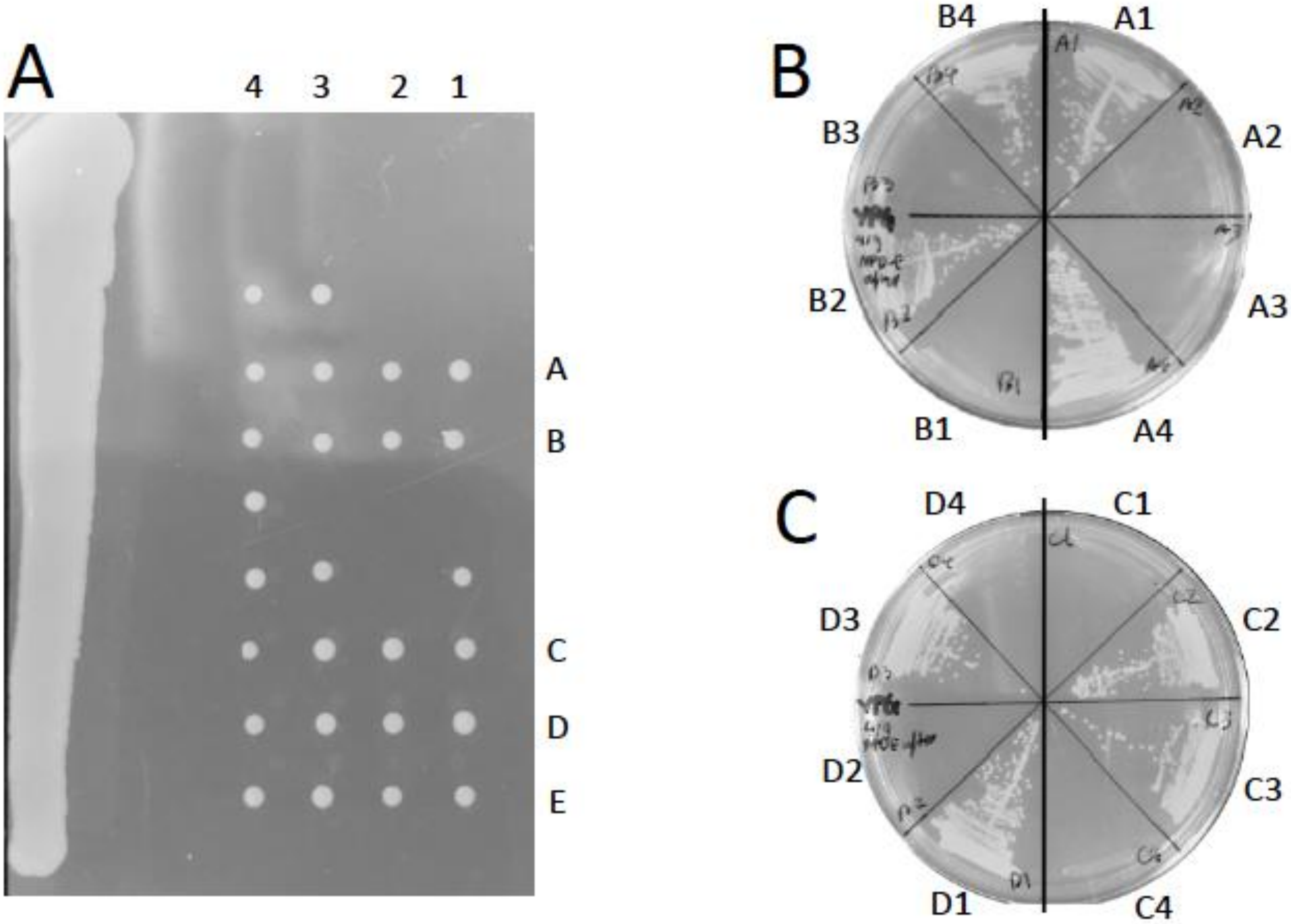

Supplement: S2 Fig — A, Tetrad dissection is shown for five sets of tetrads containing four spores (rows A-E); B, YPG growth tested for each of the colonies in rows A (A1-A4) and B (B1-B4); C, YPG growth tested for each of the colonies in rows C (C1-C4) and D (D1-D4). The 2:2 segregation of growth on YPG plate medium shown is representative of five sets of tetrads generated from the NPD-E diploid yeast strain. (PDF) [file pone.0234192.s002.pdf]
