# Supplementary material for: Intragenic suppressor mutations of the COQ8 protein kinase homolog restore coenzyme Q biosynthesis and function in Saccharomyces cerevisiae
Source: PLoS One. 2020 Jun 1;15(6):e0234192. doi: 10.1371/journal.pone.0234192 (PMC7263595; doi:10.1371/journal.pone.0234192)

S3 Fig. Test of four tetrads obtained from NPD-A diploid yeast for 2:2 segregation of growth on YPG plate medium

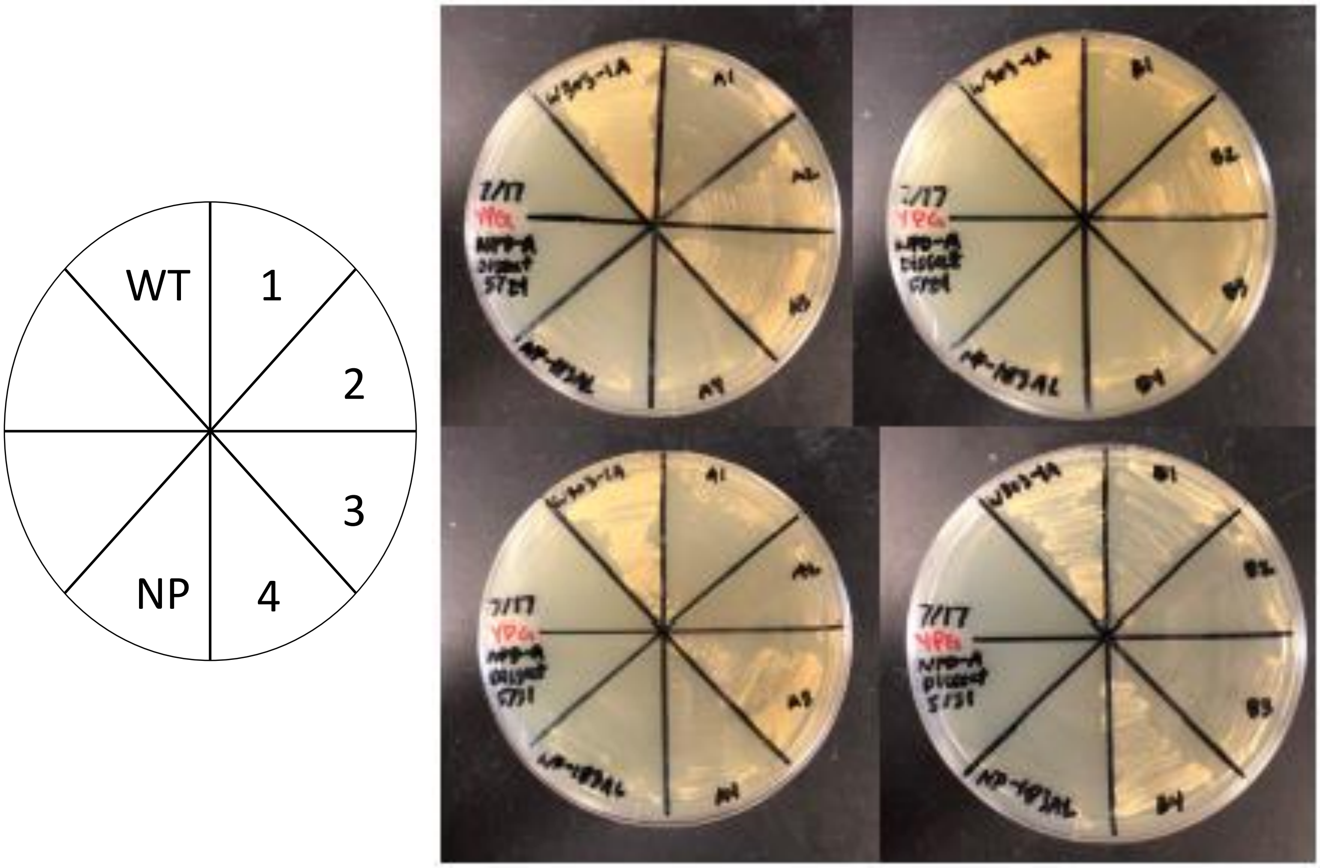

Supplement: S3 Fig — The 2:2 segregation of growth on YPG plate medium is shown for four sets of tetrads generated from the NPD-E diploid yeast strain. A-D show YPG growth tested for each of the colonies obtained from four independent tetrad dissections obtained from the sporulation of NPD-A diploid yeast strain. Also shown is the YPG positive growth of the W303-1A WT and the lack of YPG growth of the NP-183AL parental haploid strain. (PDF) [file pone.0234192.s003.pdf]
