# Supplementary figures and images for: Intragenic suppressor mutations of the COQ8 protein kinase homolog restore coenzyme Q biosynthesis and function in Saccharomyces cerevisiae
Source: PLoS One. 2020 Jun 1;15(6):e0234192. doi: 10.1371/journal.pone.0234192 (PMC7263595; doi:10.1371/journal.pone.0234192)

**S4 Fig. Unedited and uncropped full image western blots, Round 1**

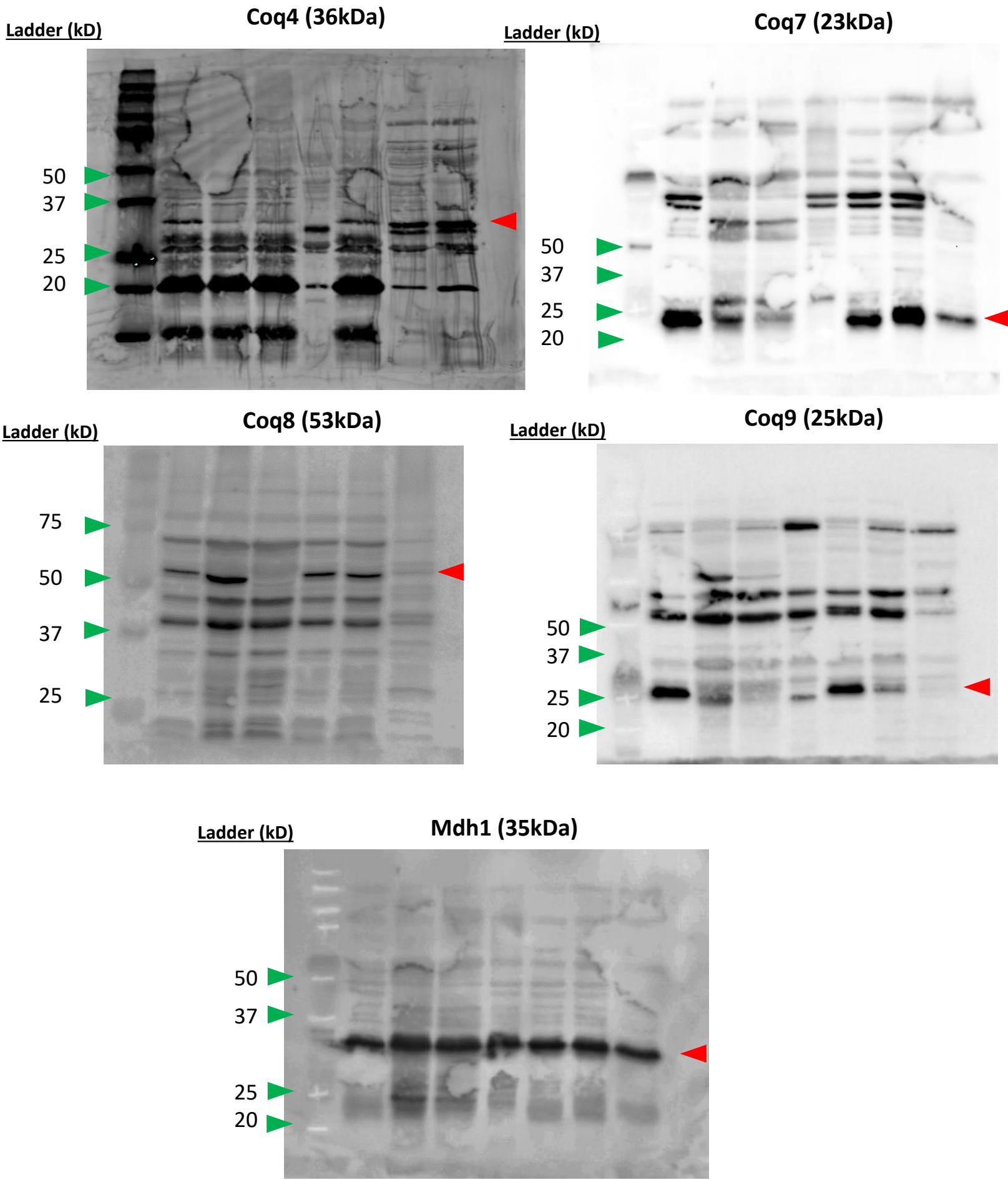

Supplement: S4 Fig — Full western blot images for Coq4, Coq7, Coq8, Coq9, and Mdh1 completed for all strains with samples of purified mitochondria. These westerns are the first of two replicates completed. The order for each lane are: ladder, WT, NP-183A, coq8Δ, coq4/7/9Δ, Rev-CL, Rev-AL, Rev-BL. Only for the Coq8 blot is the order: ladder, WT, NP-183A, coq8Δ, Rev-CL, Rev-AL, Rev-BL. The ladder molecular weight is labeled for the region flanking the respective band, which is annotated by a red arrow. (PDF) [file pone.0234192.s004.pdf]

**S5 Fig. Unedited and uncropped full image western blots, Round 2  
and used in Figure 10**

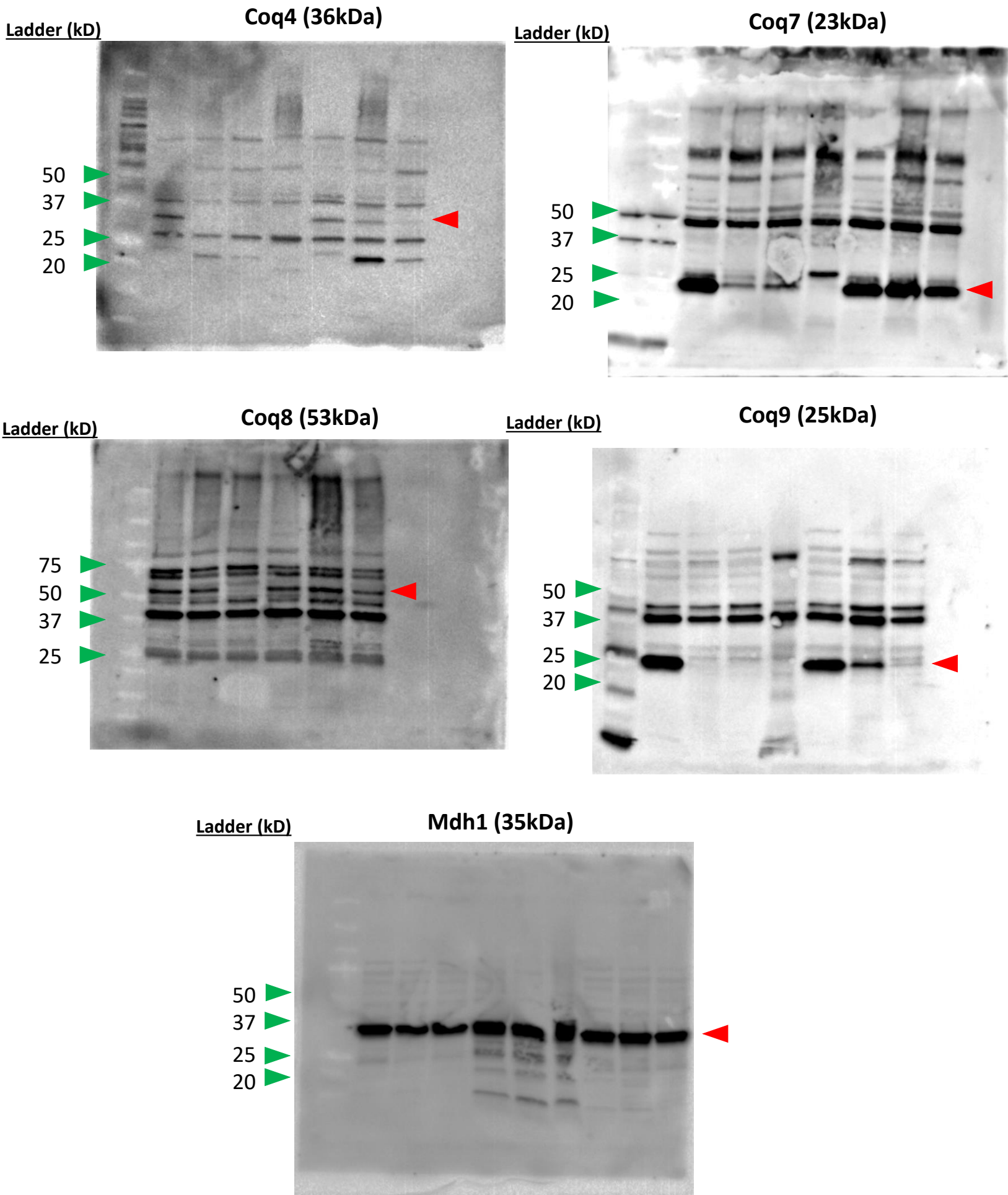

Supplement: S5 Fig — Full western blot images for Coq4, Coq7, Coq8, Coq9, and Mdh1 completed for all strains with samples of purified mitochondria. These westerns are the second of two replicates completed and were used to prepare Fig 10. The order for each lane are: ladder, WT, NP-183A, coq8Δ, coq4/7/9Δ, Rev-CL, Rev-AL, Rev-BL. Only for the Coq8 blot is the order: ladder, WT, NP-183A, coq8Δ, Rev-CL, Rev-AL, Rev-BL. The Mdh1 blot contains all the samples used for the blots, to serve as a control. The ladder molecular weight is labeled for the region flanking the respective band, which is annotated by a red arrow. (PDF) [file pone.0234192.s005.pdf]

**S7 Fig. Validation of experiment of Figure 3B, phenotype rescue as a result of plasmid expression**

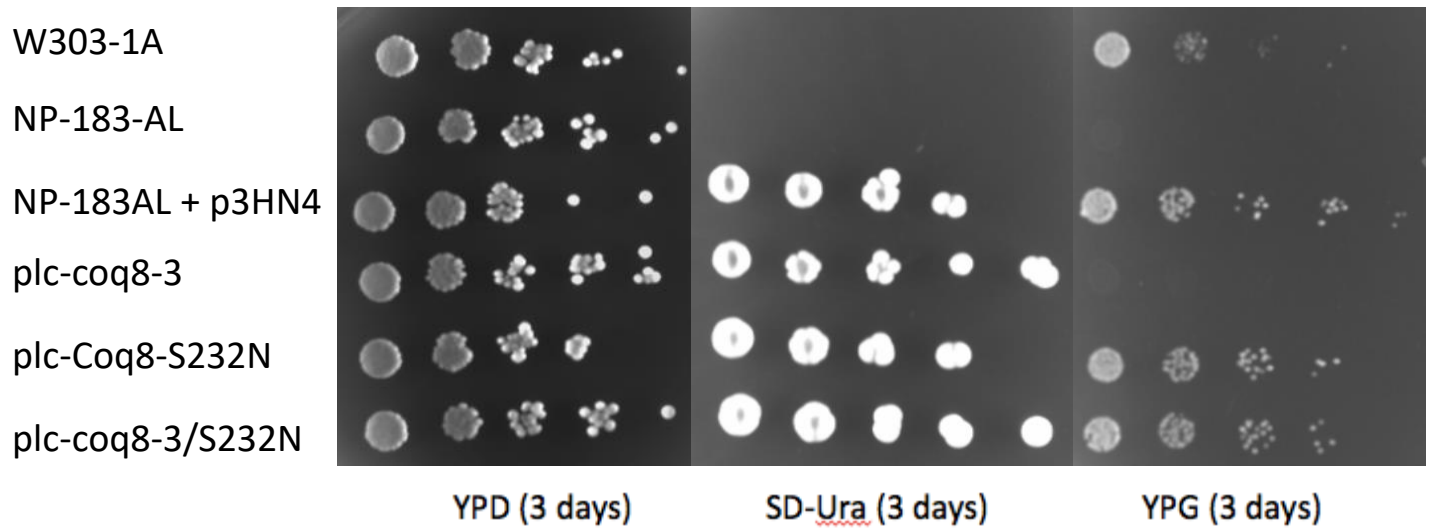

Supplement: S7 Fig — Second repetition of the phenotype rescue experiment contained in Fig 3B. (PDF) [file pone.0234192.s007.pdf]

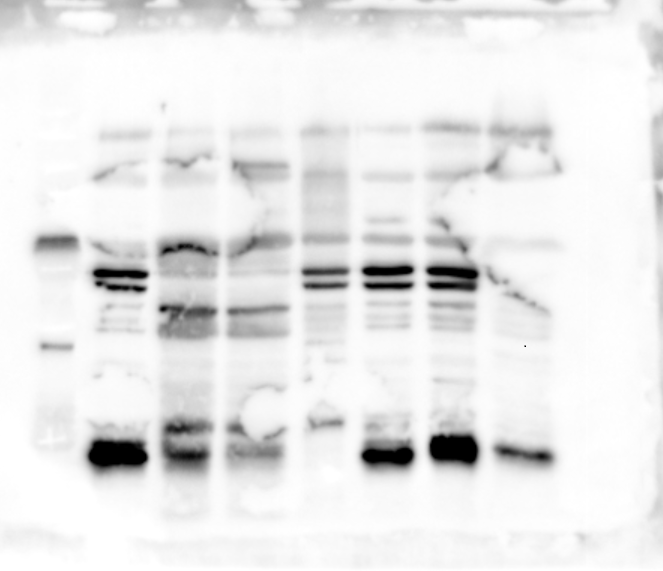

Supplement: S1 File — (ZIP) [file pone.0234192.s011.zip › raw tiff files for S figs/S5 coq7.tif]

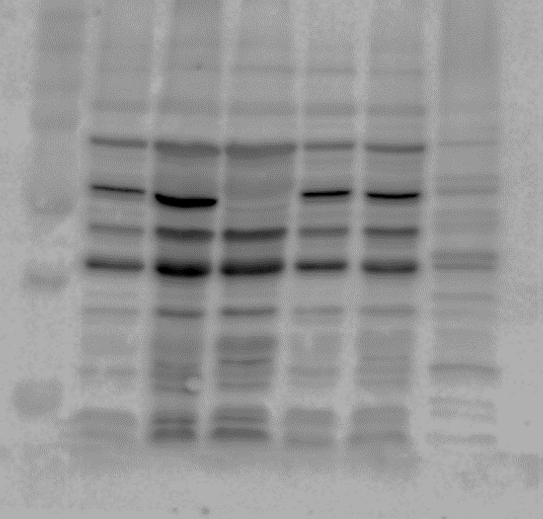

Supplement: S1 File — (ZIP) [file pone.0234192.s011.zip › raw tiff files for S figs/S5 coq8.tif]

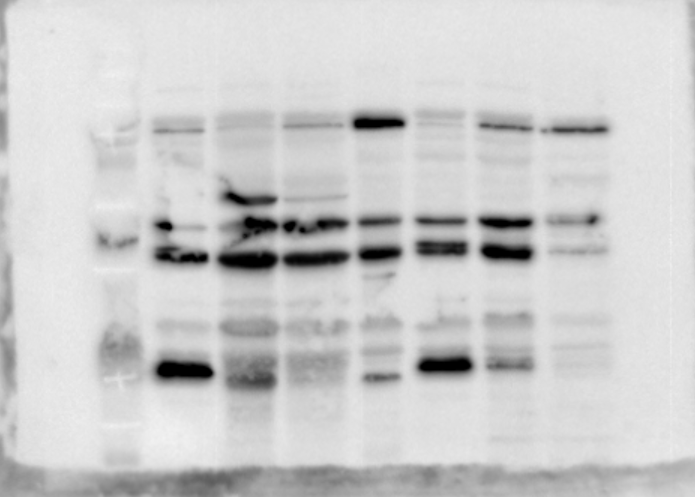

Supplement: S1 File — (ZIP) [file pone.0234192.s011.zip › raw tiff files for S figs/S5 coq9.tif]

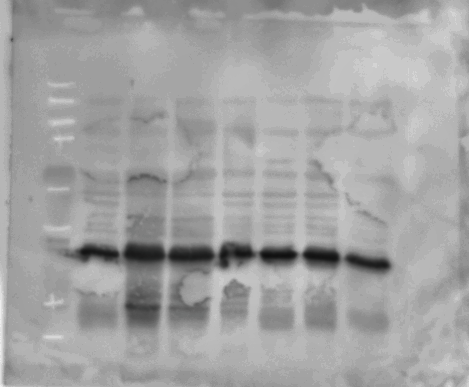

Supplement: S1 File — (ZIP) [file pone.0234192.s011.zip › raw tiff files for S figs/S5 mdh1.tif]

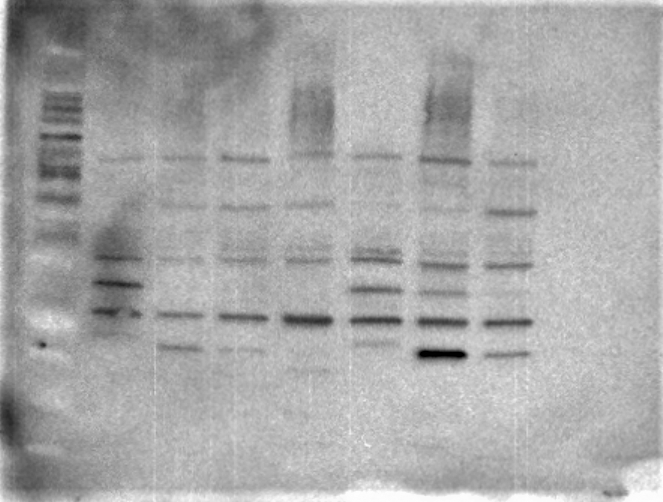

Supplement: S1 File — (ZIP) [file pone.0234192.s011.zip › raw tiff files for S figs/S6 coq4.tif]

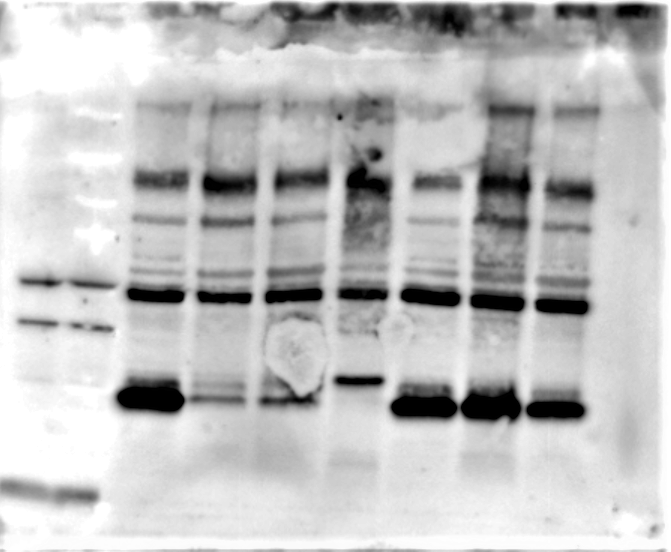

Supplement: S1 File — (ZIP) [file pone.0234192.s011.zip › raw tiff files for S figs/S6 coq7.tif]

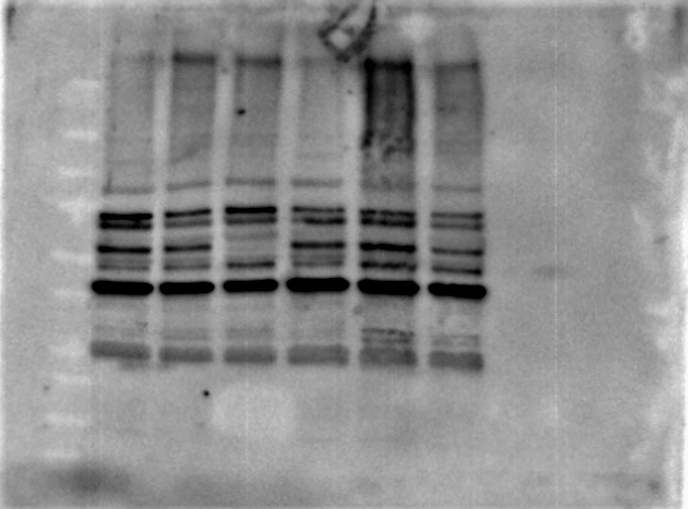

Supplement: S1 File — (ZIP) [file pone.0234192.s011.zip › raw tiff files for S figs/S6 coq8.tif]

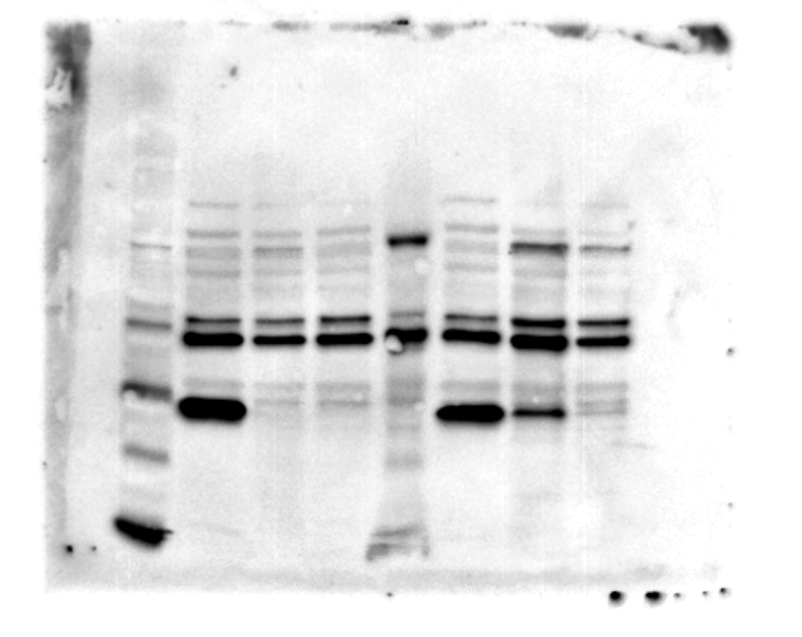

Supplement: S1 File — (ZIP) [file pone.0234192.s011.zip › raw tiff files for S figs/S6 coq9.tif]

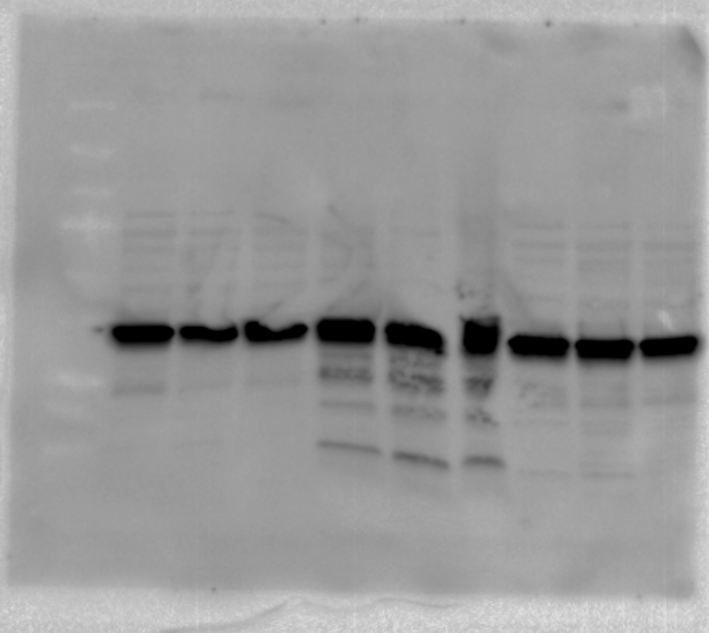

Supplement: S1 File — (ZIP) [file pone.0234192.s011.zip › raw tiff files for S figs/S6 mdh1.tif]

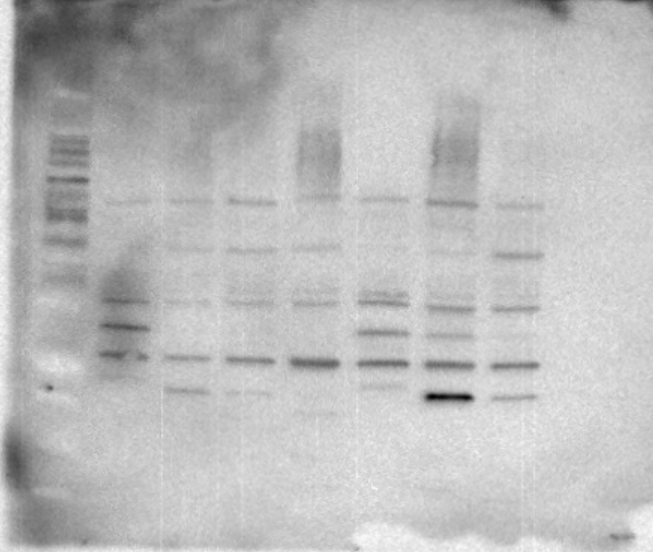

Supplement: S1 File — (ZIP) [file pone.0234192.s011.zip › raw tiff files for S figs/S7 coq4_ top panel.tif]

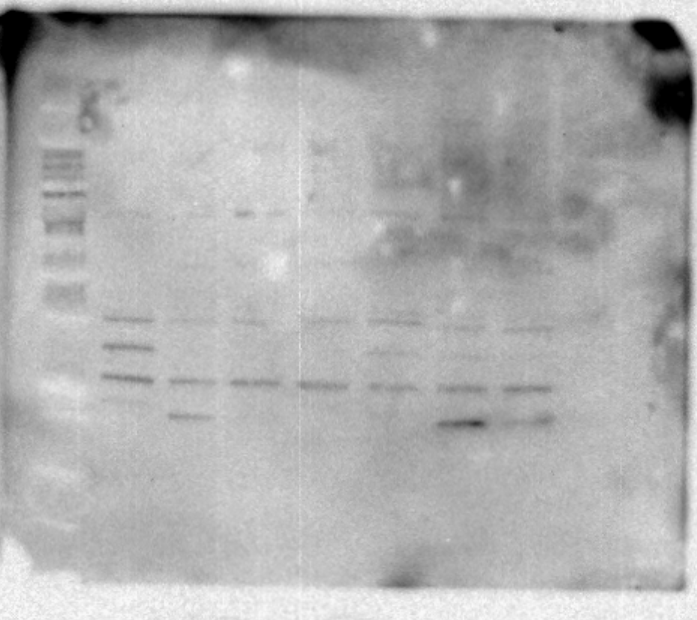

Supplement: S1 File — (ZIP) [file pone.0234192.s011.zip › raw tiff files for S figs/S7 coq4_bottom panel.tif]

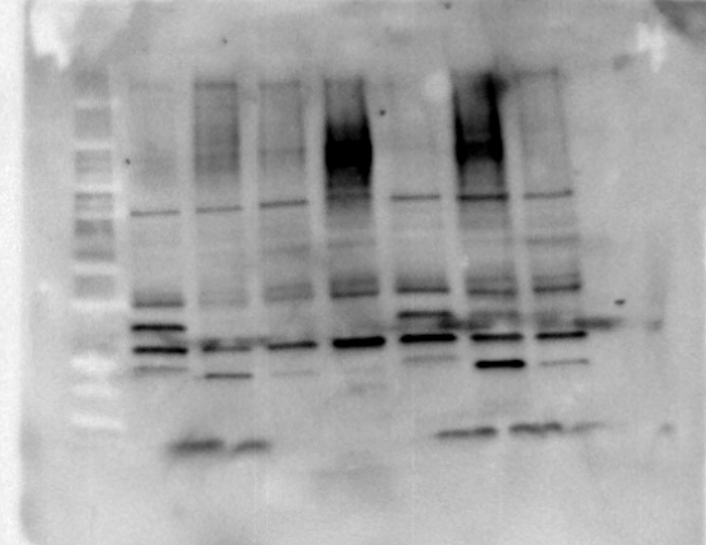

Supplement: S1 File — (ZIP) [file pone.0234192.s011.zip › raw tiff files for S figs/S7 coq4_middle panel.tif]
