# Supplementary material for: Intragenic suppressor mutations of the COQ8 protein kinase homolog restore coenzyme Q biosynthesis and function in Saccharomyces cerevisiae
Source: PLoS One. 2020 Jun 1;15(6):e0234192. doi: 10.1371/journal.pone.0234192 (PMC7263595; doi:10.1371/journal.pone.0234192)
Supplement: S6 Fig — The full image of an additional round of the Coq4 blot are shown to validate the results of S5 Fig. This is because Coq4 showed different results between S4 and S5 Figs. The results of S5 Fig appear to reflect the most consistent trends of the Coq4 polypeptide amongst all the strains. These full westerns are unedited and uncropped. The order for each lane are: ladder, WT, NP-183A, coq8Δ, coq4/7/9Δ, Rev-CL, Rev-AL, Rev-BL. Note that the background species are not uniform between the lanes for each strain, probably due to the polyclonal sera used in these experiments. (PDF) [file pone.0234192.s006.pdf]

S6 Fig. Coq4 blot validated again to affirm results of the respective blot from S5 Fig.

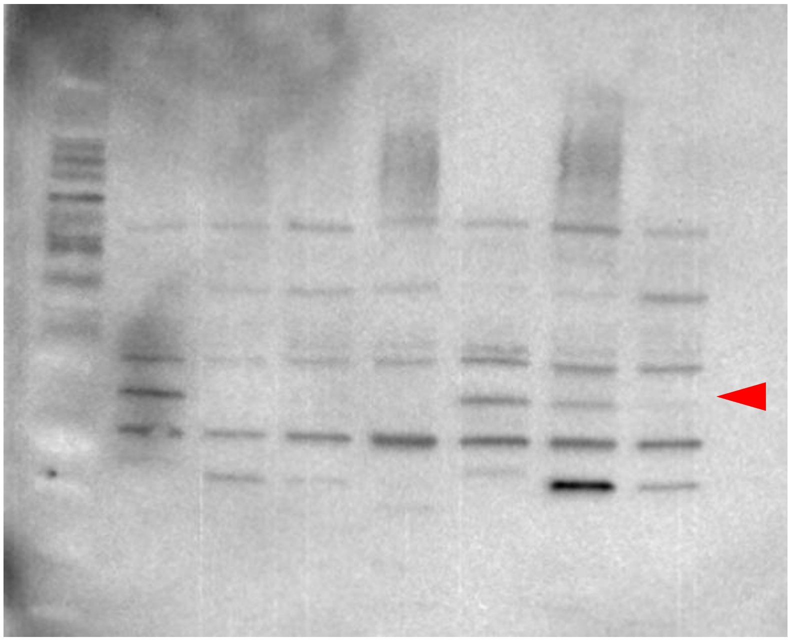

*This is the one used in the revised panel

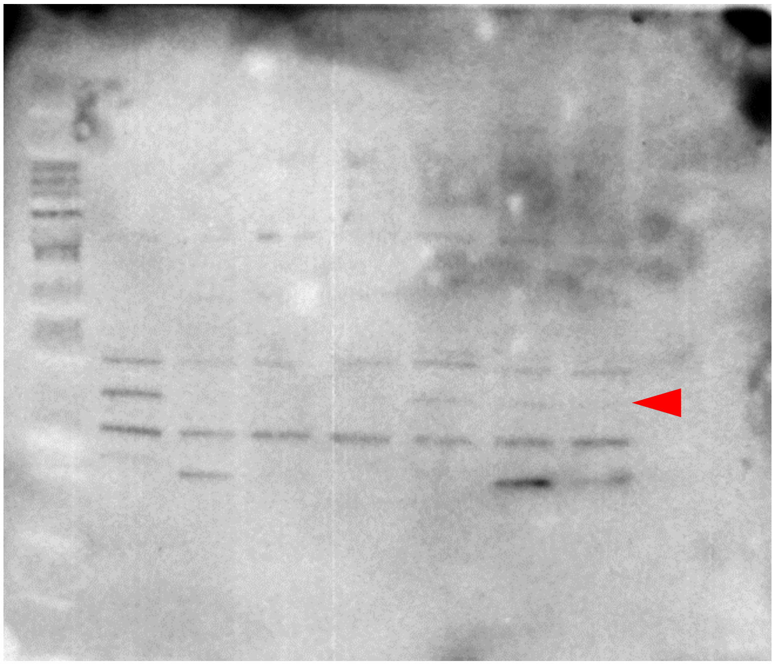

*This to validate the top result
