# Supplementary material for: Intragenic suppressor mutations of the COQ8 protein kinase homolog restore coenzyme Q biosynthesis and function in Saccharomyces cerevisiae
Source: PLoS One. 2020 Jun 1;15(6):e0234192. doi: 10.1371/journal.pone.0234192 (PMC7263595; doi:10.1371/journal.pone.0234192)

S8 Fig. Validation of experiment in Fig 2, plate dilution assay and growth on YPD and YPG plate medium

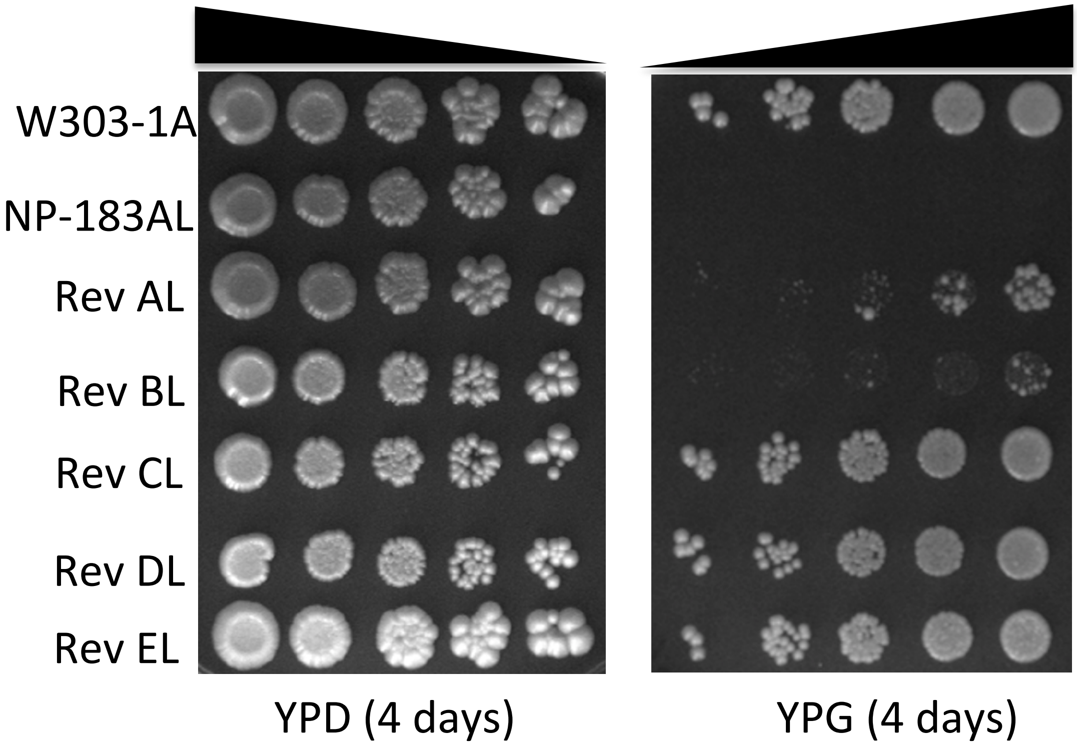

Supplement: S8 Fig — Second repetition of the plate dilution assessment of growth present in Fig 2. (PDF) [file pone.0234192.s008.pdf]
