# Supplementary material for: Intragenic suppressor mutations of the COQ8 protein kinase homolog restore coenzyme Q biosynthesis and function in Saccharomyces cerevisiae
Source: PLoS One. 2020 Jun 1;15(6):e0234192. doi: 10.1371/journal.pone.0234192 (PMC7263595; doi:10.1371/journal.pone.0234192)

S8 Fig. Validation of experiment in Fig 2, plate dilution assay and growth on YPD and YPG plate medium

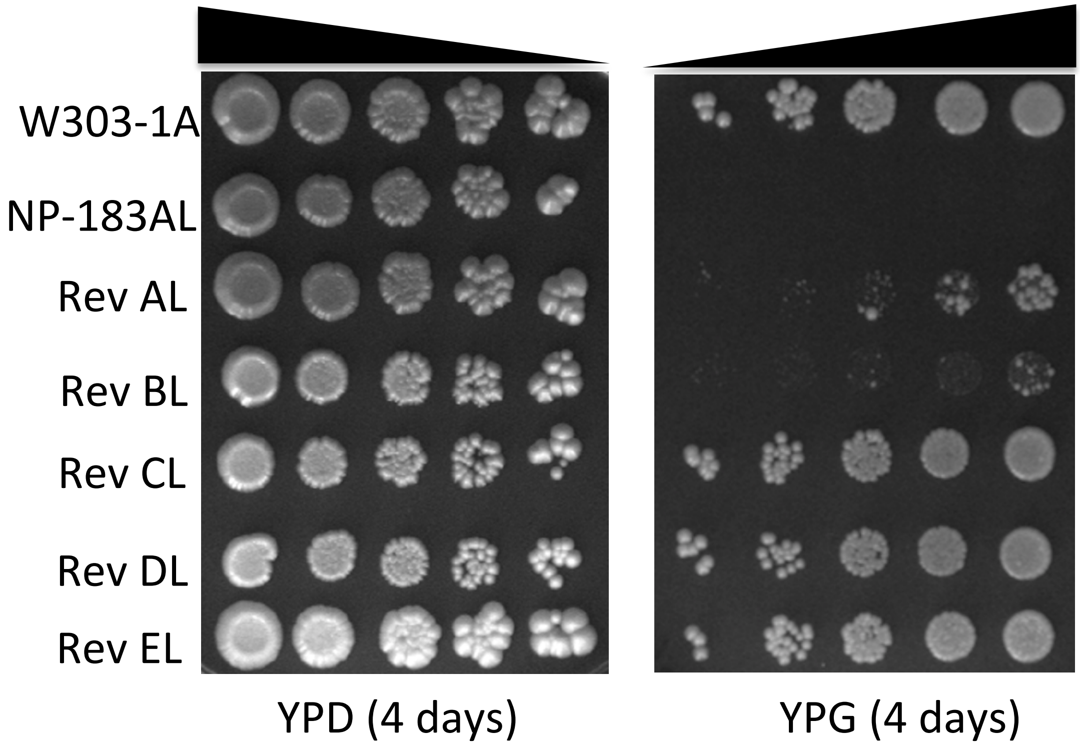

S9 Fig. Validation of experiment in Fig 3A, plate dilution assay and growth on YPD and YPG plate medium of diploid strains

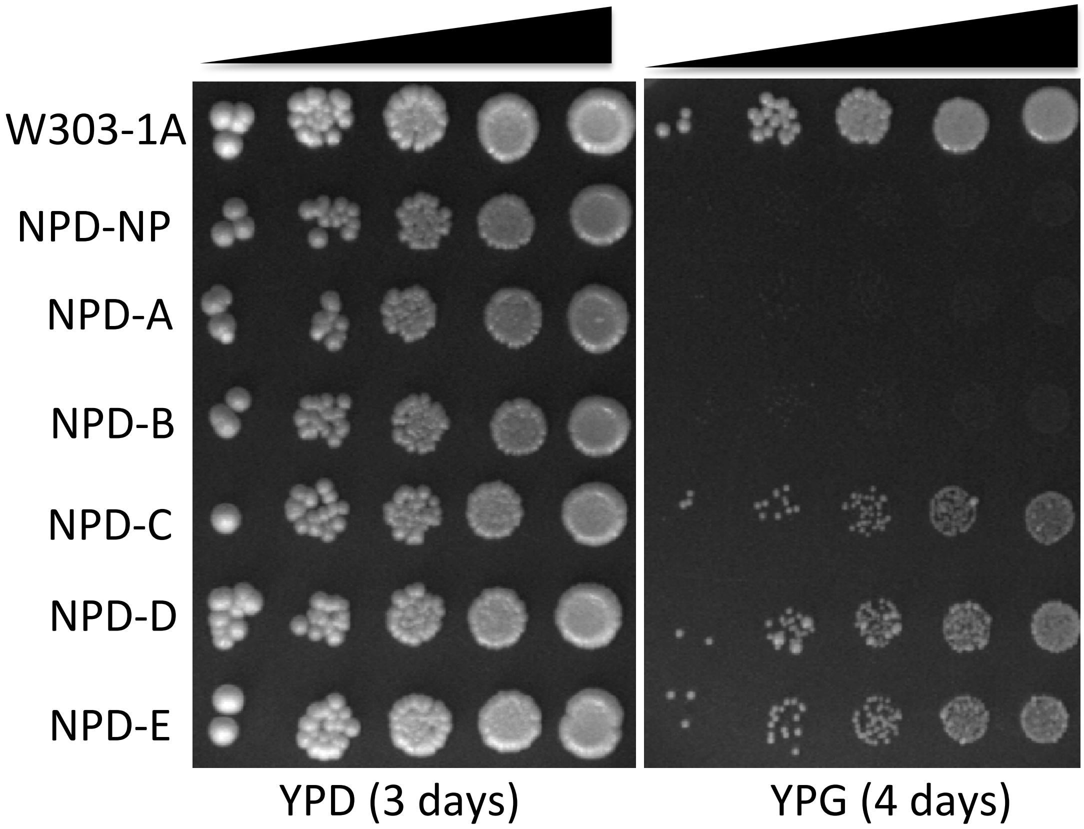

Supplement: S9 Fig — Second repetition of the plate dilution assessment of growth presented in Fig 3A. (PDF) [file pone.0234192.s009.pdf]
