# Supplementary material for: Intragenic suppressor mutations of the COQ8 protein kinase homolog restore coenzyme Q biosynthesis and function in Saccharomyces cerevisiae
Source: PLoS One. 2020 Jun 1;15(6):e0234192. doi: 10.1371/journal.pone.0234192 (PMC7263595; doi:10.1371/journal.pone.0234192)
Supplement: S1 Raw images — (PDF) [file pone.0234192.s010.pdf]

S5 Fig. Unedited and uncropped full image western blots, Round 1

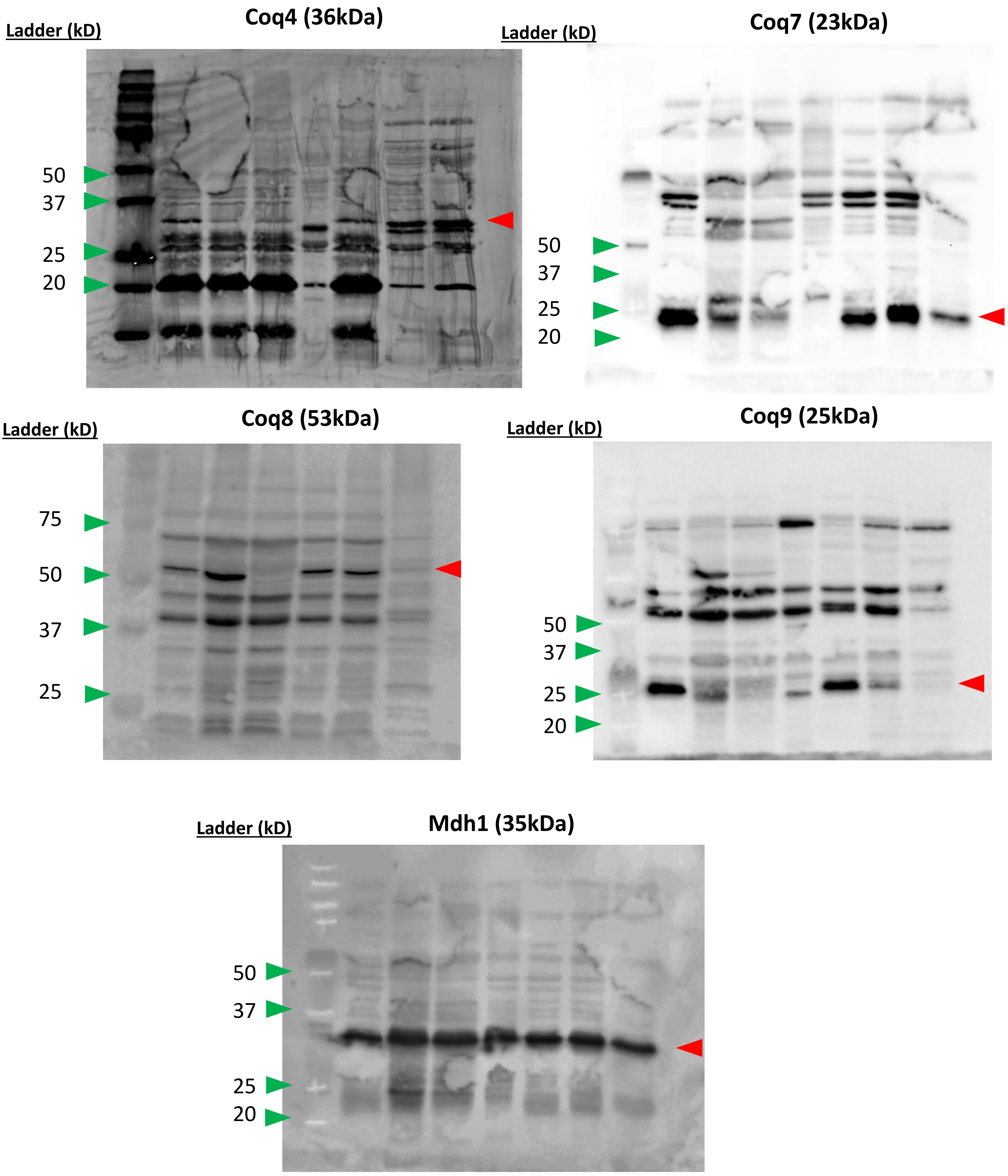

S6 Fig. Unedited and uncropped full image western blots, Round 2 and used in Figure 10

Ladder (kD) **Coq4 (36kDa)**

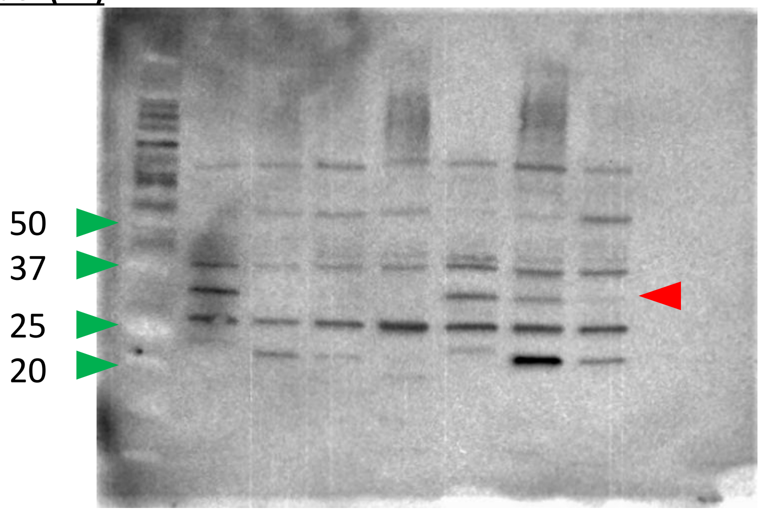

Ladder (kD) **Coq7 (23kDa)**

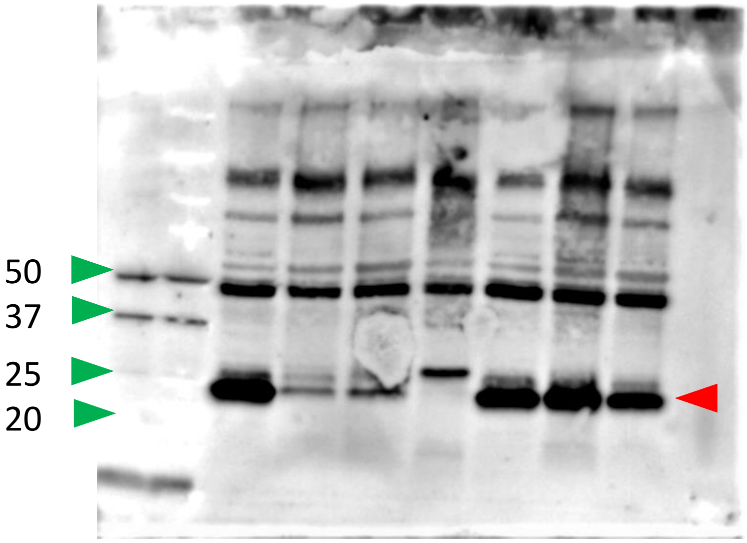

Ladder (kD) **Coq8 (53kDa)**

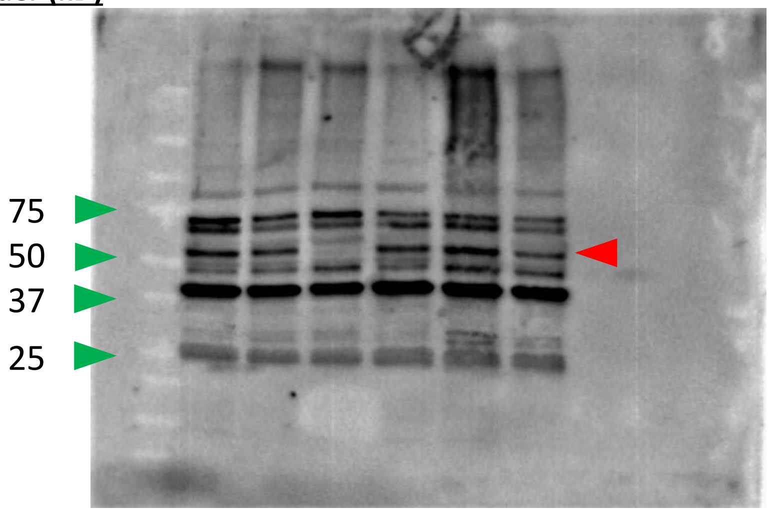

Ladder (kD) **Coq9 (25kDa)**

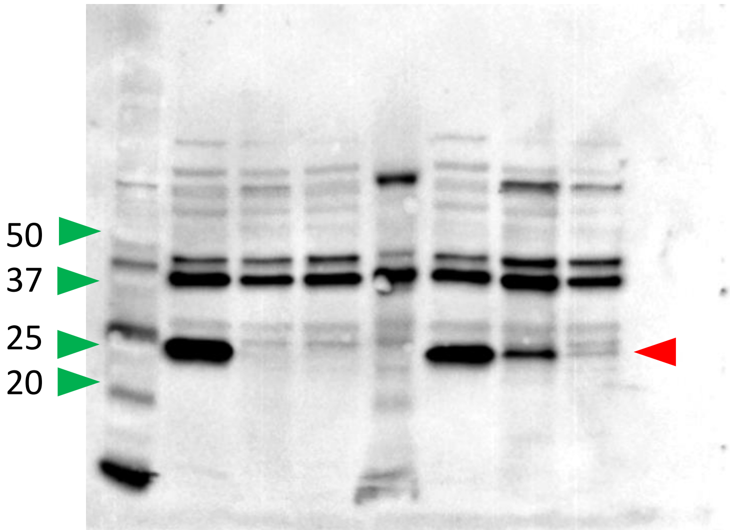

Ladder (kD) **Mdh1 (35kDa)**

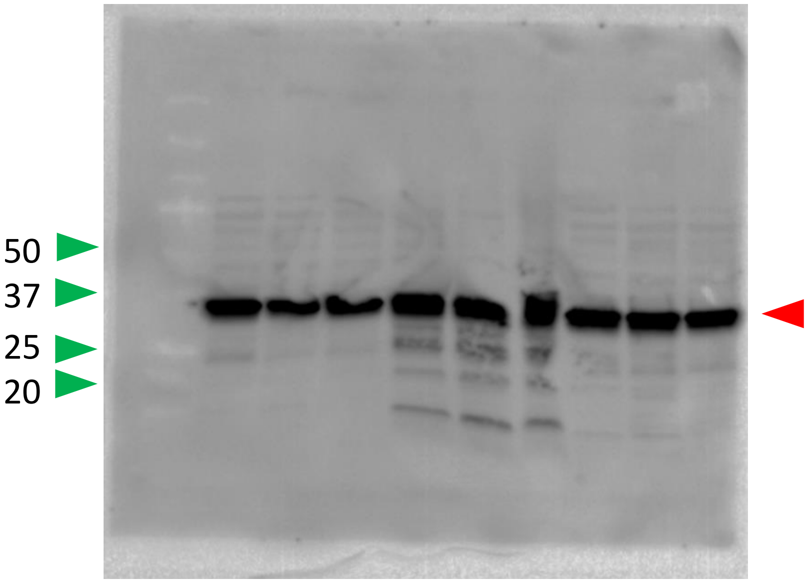

S7 Fig. Coq4 blot validated twice more to affirm results of the respective blot from S6 Fig.

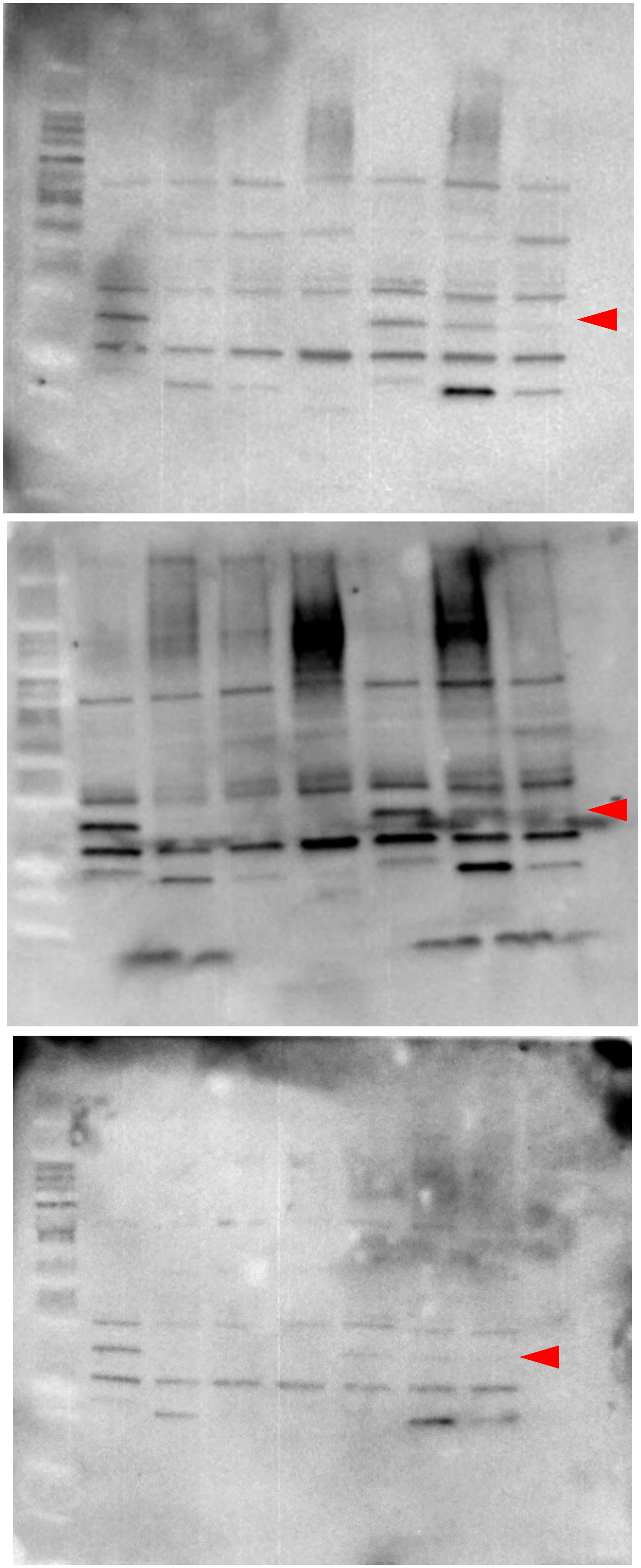

*This is the one used in the revised panel

*These two are to validate the top result
